# A Scheme of Bandwidth Demands Capture and Resource Allocation Based on Martingale Theory Under Statistical End-to-End Delay QoS Constraints

**DOI:** 10.3390/s26216692

**Published:** 2026-10-22

**Authors:** Baozhu Yu, Dingjie Wang, Tingting Sui

**Affiliations:** 1 School of Information Science and Engineering, Shenyang Ligong University, Shenyang 110159, China; yubz22@sylu.edu.cn; 2 School of Emergency Equipment (Emergency Design Center), North China Institute of Science and Technology, Sanhe 065201, China

**Keywords:** QoS, end-to-end delay, martingales, resource allocation

## Abstract

Statistical end-to-end (E2E) delay quality-of-service (QoS) provisioning is pivotal for ultra-reliable low-latency communication (URLLC) in 5G/B5G networks. Based on martingale theory, we propose an E2E delay analysis framework for paths with heterogeneous service processes. Martingales related to the arrival and service processes are constructed, and bias scale functions are defined via martingale parameters to characterize the statistical features of the accumulated arrival and service. A service index is introduced to model the service capability of the tandem queuing system in the martingale domain. By combining the service index with the Doob maximum inequality, a tight upper bound of the E2E delay violation probability is derived. For the parallel transmission pattern constructed for URLLC traffic, a bandwidth demand capture and reliability decomposition (BDC-RD) scheme is designed, which obtains the bandwidth requirements of all involved nodes and decomposes the reliability requirement across different E2E paths. Guided by the martingale-based upper bound, an optimization problem for QoS provisioning is formulated and solved by the particle swarm optimization (PSO) algorithm, achieving the decoupling between the statistical QoS requirements and the desired bandwidth of each node. In the radio access network, a physical-layer resource allocation algorithm is proposed under the bandwidth demand constraints, and a closed-form solution of the power consumption is derived. The effectiveness of the proposed BDC-RD scheme and the physical-layer resource allocation algorithm is verified. The PSO solutions are shown to be close to the global optimum by comparison with the global-optimum lower bound obtained by the branch-and-bound search. The superiority of the theoretical result on the delay violation probability is demonstrated.

## 1. Introduction

Ultra-Reliable Low Latency Communications (URLLCs) are highly anticipated services in 5G networks poising to bring end-to-end (E2E) transmission of unprecedented quality of service (QoS) requirements. The data traffic generated from the URLLCs, such as the telemedicine, factory and process automation, requires strict reliability provisioning related to E2E delay, which desires the E2E delay to be within 1–10 ms with a reliability of 99.999% or even 99.9999% [1]. According to 3GPP, time-critical applications require an E2E delay of no more than 1 ms, decomposed into radio processing and transmission (0.25 ms), queuing and frame alignment (0.25 ms), and core network transport (0.5 ms) [2]. The QoS demands induced by service characteristics have a significant impact on the current network architecture. The huge bandwidth cost must be incurred to carry out such traffic, which challenges the scarce licensed resources. Meanwhile, the E2E delay QoS requirement is a statistical index in a long time scale. The coupling between network resource configuration at small time granularity and statistical reliability guarantees remains ambiguous. To utilize the network resource efficiently, a precise theoretical analysis framework of the statistical E2E delay performance is presented, which should expose the hidden influence mechanism of the data traffic characteristics and the heterogeneous transmission schemes of the involved nodes in the E2E path on the E2E delay clearly. The probability of the E2E delay violating the threshold, termed the E2E delay violation probability, is essentially the complementary cumulative distribution function of the E2E delay. From the perspective of the queuing system, the E2E delay arises from the imbalance between the arrival load and the service provisioning along the E2E path and depends on the randomness of the arrival process, the fading characteristics of the wireless channels, the access mechanisms in the radio access network (RAN), and the scheduling mechanisms of the forwarding nodes in the transport/core networks. In essence, the E2E delay is the product of multiple random processes with various statistical characteristics entangled with each other. Due to the limitations of theoretical analysis tools, we can only capture the average characteristics of the E2E delay and the upper bounds of the E2E delay violation probability. Existing approaches to E2E delay analysis can be broadly categorized into tandem queuing theory, stochastic network calculus (SNC) [3], the effective bandwidth/effective capacity (EB/EC) framework [4,5,6], and martingale theory. Tandem queuing theory characterizes the average delay but cannot capture the rare delay violation events that are critical for URLLC. SNC provides delay bounds through convolutions, but the union bound inequality often leads to loose bounds [3]. The EB/EC framework, rooted in large deviation theory, offers a statistical delay bound without explicit assumptions on the arrival and service processes, but it is relatively conservative for bursty traffic [4,5,6]. Martingale theory has shown advantages in precisely evaluating the delay performance of single-node and multi-hop systems, but existing E2E frameworks commonly assume homogeneous service processes across nodes, which limits their applicability to heterogeneous networks. The interplay between statistical E2E delay QoS provisioning and fine-grained network resource configuration remains elusive. The statistical QoS requirements are inherently probabilistic and derived from long-term observations, necessitating adaptive resource allocation in response to dynamic network conditions. The work in [7] introduced a bandwidth abstraction and link rate instantiation mechanism for delay-constrained access slices, decoupling reliability from bandwidth demand via the EB/EC framework. However, the proposed scheme falls short of extending to E2E delay QoS provisioning scenarios. In particular, this paper considers a hot-standby transmission mode, where an E2E parallel transmission system is established for each user to enhance QoS guarantees. Under this scenario, the core problem is how to decouple the E2E delay QoS requirement as the corresponding bandwidth demands for each node involved in the parallel transmission system. In this paper, based on martingale theory, we construct a precise E2E delay performance analysis framework for tandem queuing systems with heterogeneous service processes. By defining martingales related to the arrival and service processes, along with bias scale functions and a service index, a tight upper bound of the E2E delay violation probability is derived. Guided by this upper bound, we design a bandwidth demand capture and reliability decomposition (BDC-RD) scheme for a backup transmission pattern, which decouples the statistical E2E delay QoS requirements into the demanded service rates of each node. Under the bandwidth demand constraints, a physical-layer resource allocation algorithm is further proposed to achieve the E2E delay QoS provisioning. The contributions in this paper can be concluded as follows.

(1) A martingale-based E2E delay analysis framework is proposed. Leveraging martingale theory and stochastic network calculus (SNC), we construct an E2E delay analysis framework for paths with heterogeneous service processes. Martingales related to the arrival and service processes are defined, and bias scale functions and a service index are introduced to characterize the statistical features of the accumulated arrival and service and to model the service capability of the tandem queuing system in the martingale domain. By combining the Doob maximum inequality with the service index, a tight upper bound of the E2E delay violation probability is derived.

(2) A bandwidth demand capture and reliability decomposition (BDC-RD) scheme is proposed. A hot-backup transmission mode is adopted, and a parallel system is constructed for the E2E traffic. Based on reliability theory and the martingale-based upper bound, the E2E delay reliability of the parallel system is modeled in the martingale domain. A BDC-RD scheme is proposed, which decomposes the statistical E2E delay QoS requirements into the demanded service rates of each node and the reliability requirements of each E2E path. An optimization problem minimizing the total bandwidth provisioning is formulated and solved by the particle swarm optimization (PSO) algorithm, yielding the bandwidth demand of each node while accounting for the heterogeneity of service processes.

(3) A physical-layer resource allocation algorithm is designed. Under the bandwidth demand constraints in the radio access network (RAN), an optimization problem for transmission power configuration is constructed. Using the Lagrange gradient framework, a closed-form solution of the minimum allocated power for each E2E path is derived, which achieves the mapping from the statistical E2E delay QoS requirements to the allocated resources at a small time granularity. Simulation results verify the effectiveness of the proposed BDC-RD scheme and the physical-layer resource allocation algorithm, demonstrate the superiority of the martingale-based bound over state-of-the-art results, and confirm that the PSO solutions are very close to the global optimum.

The rest of this paper is organized as follows. In Section 2, the related work is reviewed. In Section 3, the network scenario and the tandem queuing system are described. In Section 4, the martingale-based E2E delay performance analysis framework is introduced. In Section 5, the BDC-RD scheme is designed. And the physical layer resource allocation algorithm is proposed in Section 6. Simulations and results analysis are shown in Section 7. Finally, we conclude this paper in Section 8. The notations mainly used in the paper are summarized in Table 1. And the involved abbreviations are shown in Table 2.

## 2. Related Work

Tandem queuing theory provides a fundamental framework for modeling the E2E transmission process through multiple service stages. Deng et al. [8] modeled task uploading, forwarding, and computing in UAV-assisted mobile edge computing as a three-stage tandem queue and derived an analytical E2E delay bound. Zhao et al. [9] modeled packet encoding and symbol transmission in URLLC systems as tandem queues and analyzed the minimum average delay under reliability and power constraints. These studies show that tandem queuing theory is effective in characterizing the average E2E delay and supporting system design. However, it mainly captures the average delay behavior and cannot characterize the rare delay violation events that are critical for URLLC. SNC has been increasingly applied to statistical E2E delay characterization in emerging communication networks. Cui et al. [10] evaluated the E2E delay violation probability in industrial IoT networks within an SNC framework. Miao et al. [11] developed an SNC-based analytical model for vehicular edge computing and derived a worst-case E2E delay bound. SNC was also applied to xURLLC systems to characterize statistical delay guarantees [12] and to guide a learning-based dynamic network slice resizing strategy [13]. These studies demonstrate the applicability of SNC to statistical delay analysis and delay-aware resource management. However, the union bound inequality commonly used in SNC often leads to loose delay bounds, which may result in inefficient resource allocation. The EB/EC framework has also been incorporated into delay-aware communication and resource-management problems. Zeng et al. [14] investigated statistical delay QoS in uplink non-orthogonal multiple access (NOMA) systems and developed effective-capacity-based power allocation schemes. NOMA has also been investigated for massive machine-type communications (mMTC), where sparse code multiple access (SCMA) can be employed to support massive connectivity [15]. Prakash et al. [16] developed an effective-bandwidth-based stochastic delay model for dual-connectivity systems and optimized uplink traffic allocation. More recently, Li et al. [17] employed effective capacity to characterize the reliability–latency–rate tradeoff in low-latency communications with finite-blocklength coding. These studies demonstrate the applicability of the EB/EC framework to statistical delay characterization and resource allocation. However, the EB/EC framework is rooted in large deviation theory and often yields a relatively conservative bound, especially for bursty traffic and small buffer backlog. Martingale theory has demonstrated notable advantages in precisely evaluating the delay performance of single-node systems. A series of studies [3,18,19] proposed a systematic approach to deriving upper bounds on the delay violation probability through tailored exponential martingale constructions, and the resulting bounds were shown to outperform existing results by several orders of magnitude [18]. Hu et al. [20] extended the martingale framework to multi-hop systems with Markov-modulated arrival and service processes, deriving an upper bound of the E2E delay violation probability. This framework was subsequently adopted in [21,22,23,24] for E2E delay analysis in specific application contexts, including virtual reality services over Terahertz networks [21] and edge computing [22]. More recently, Yan et al. [25] developed a martingale-based E2E delay analysis for multi-hop wireless links, where arrival and service martingales were constructed in the SNR domain. These studies demonstrate the extension of martingale-based delay analysis from single-node systems to E2E multi-hop scenarios. However, the existing E2E frameworks commonly assume that the service processes at all nodes are statistically homogeneous, which limits their applicability to networks with heterogeneous services. Martingale-based delay analysis has also been incorporated into practical QoS provisioning and resource-management problems. Liu et al. [26] constructed queue-length and delay martingales for bursty traffic in 5G wireless access networks and used the resulting delay reliability bound to guide bandwidth abstraction and transmission power allocation. Similar martingale-based formulations have been adopted for radio resource orchestration under delay violation constraints in 6G O-RAN systems [27] and for statistical delay analysis of heterogeneous aggregate traffic, where the obtained delay bound was further exploited for bandwidth estimation and wireless resource allocation [28]. These studies demonstrate that martingale-based delay bounds can support practical QoS provisioning and resource allocation. However, most of them focus on a single path and do not address reliability decomposition and bandwidth demand capture in a parallel transmission system.

## 3. The Network Model and the Queuing System

**Figure 1:**
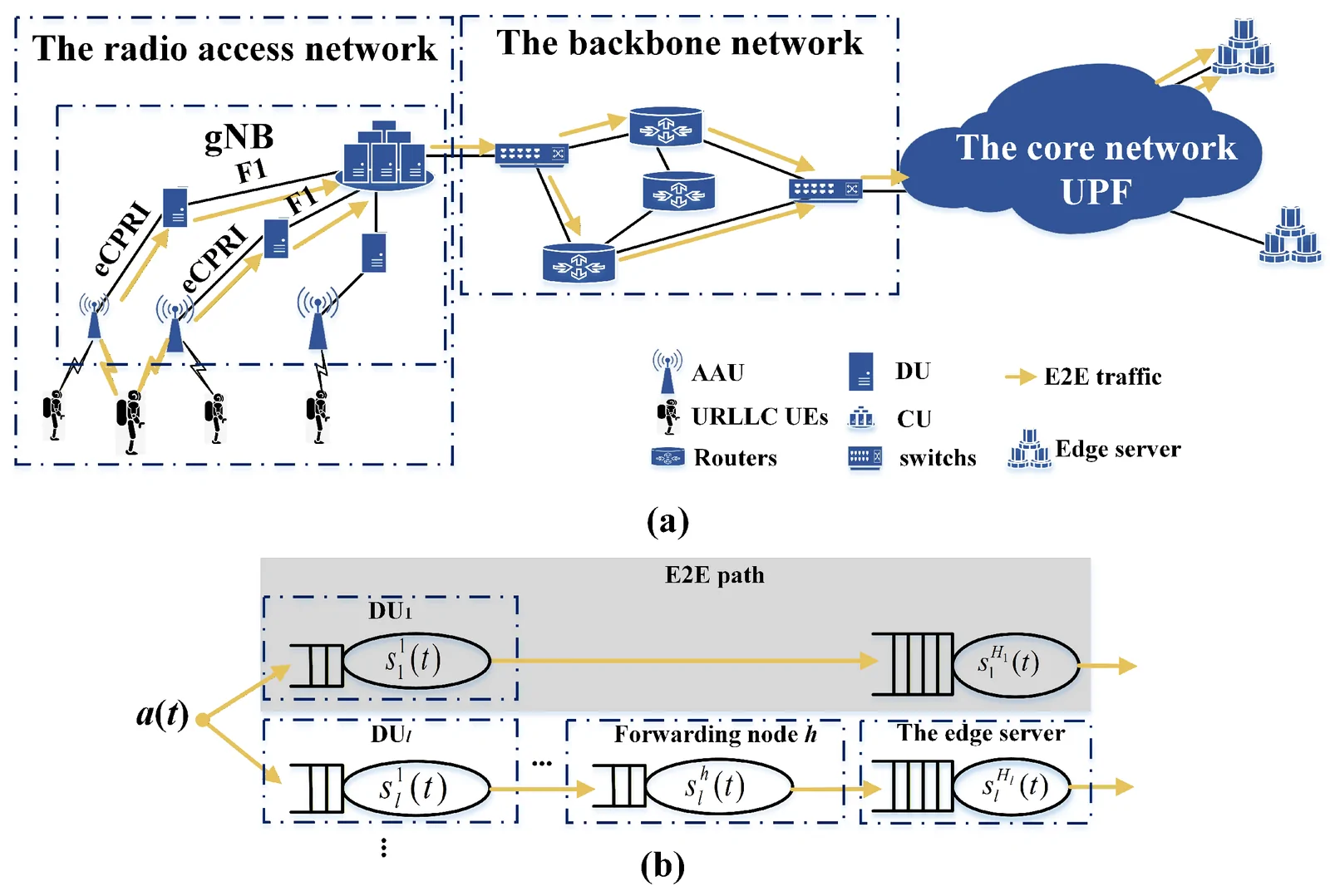
The scenario diagram. (**a**) the E2E network architecture; (**b**) the parallel queuing system.

We consider an uplink scenario of the target user equipment (UE), as shown in Figure 1a. The considered network follows the 5G/B5G architecture, which consists of three main parts: the radio access network (RAN), the transport/backbone network, and the core network. In the RAN, we consider the next-generation NodeB (gNB), which in 5G is logically split into a centralized unit (CU) and a distributed unit (DU) and is connected to an active antenna unit (AAU). The AAU communicates with the UE over the wireless link and is connected to the DU via the fronthaul link (CPRI/eCPRI). The DU handles real-time baseband processing, including the MAC-layer random access procedure, and is connected to the CU via the F1 interface. The CU handles non-real-time functions such as RRC and PDCP. Both the CU and the DU can be virtualized and run as software on a generic server pool, referred to as the virtualized baseband unit (vBBU) or O-Cloud. The connection between the CU and the backbone network is referred to as backhaul. The backbone network connects the RAN to the core network and consists of routers and switches. The user plane function (UPF) is the only core network function considered, which is responsible for user-plane packet routing and forwarding. An edge server is deployed at the edge of the core network to host the target application. The transport network nodes (routers and switches) and the UPF are collectively referred to as forwarding nodes.

To provision the E2E QoS requirement, a backup transmission pattern is adopted. The data packets are duplicated, and each copy is transmitted over a separate E2E path. Multi-path routing enhances network availability by selecting failure-independent paths [29]. The paths are topologically disjoint, thereby avoiding the situation where a single point of failure causes all paths to fail simultaneously. It is assumed that the multiple paths in the parallel transmission system are node-disjoint. The E2E paths associated with the UE are constructed as a parallel transmission system. The set of these E2E paths is denoted as L={1,⋯,L}. The E2E path l,l∈L includes multiple nodes, the set of which is $H_{l}=\left\{1,2,\cdots ,H_{l}\right\}$.

In E2E path l,l∈L, node 1 corresponds to the specific DU that serves the target UE on this path, denoted as ${\mathrm{DU}}_{l}$. The target UE accesses ${\mathrm{DU}}_{l}$ through the associated AAU. Since the parallel paths are node-disjoint, different paths may be associated with different DUs and AAUs. The random access occur at ${\mathrm{DU}}_{l}$. Based on the Shannon theorem, the achievable transmission rate from the target UE to the AAU associated with E2E path *l* at time slot *t*, which is denoted as $R_{l}^{1}\left(t\right)$, can be written as

(1) $$\begin{array}{c} R_{l}^{1}\left(t\right)=W\log_{2}\left(1+\frac{p_{l}^{1}\left(t\right){h_{l}^{1}}^{2}}{\sigma^{2}}\right)\left[\mathrm{bits}/s\right], \end{array}$$

where *W* is the bandwidth allocated to the DU. $p_{l}^{1}\left(t\right)$ is the transmission power and $\sigma^{2}$ is the noise power. $h_{l}^{1}=\sqrt{\left(\gamma_{l}^{1}\tau \left(d_{l}^{1}\right)\right)}\iota_{l}^{1}$, where $\gamma_{l}^{1}$ accounts for the shadowing fading and $\tau (d_{l}^{1})$ is the path loss between the UE and the DU belonging to E2E path *l*. $d_{l}^{1}$ is the distance. $\iota_{l}^{1}∼CN\left(0,1\right)$ denotes the small-scale fading and $\iota_{l}^{1}$ is assumed to remain unchanged in one time slot. The maximum transmission power of the UE is given as $p^{\max}$, which is limited as

(2) $$\sum_{l\in L}p_{l}^{1}\left(t\right)\leq p^{\max}.$$

In the forwarding nodes and the edge server, the traffic on the E2E path *l* is served with a rate $R_{l}^{h}\left(t\right)$ bits/s in node $h,h\in H_{l}\setminus \left\{1\right\},l\in L$, which is a constant.

The network model in Figure 1a is an abstract representation of the 5G/B5G E2E path, focusing on the queuing and resource allocation aspects relevant to the E2E delay analysis. For E2E path *l*, it can be modeled as a tandem queuing system as shown in Figure 1b. The arrival process describes the generation of data traffic at the UE. Since the delay is defined from the perspective of time slots, the arrival rate and service rates are sampled in the units of packets/slot instead of bits/s. The arrival process is denoted as {a(t),t≥0}, where a(t) is the number of generated packets at time slot *t*. Due to the packet duplication, $a\left(t\right)=a_{l}\left(t\right),l\in L$. We assume that {a(t),t≥0} possesses the i.i.d. statistical property.

In the RAN, the random access and queuing occur at the DU part. We consider that the URLLC UE adopts the ALOHA-like random access pattern, which is able to offer low queuing delay and access delay in the case that the successful access probability of the target UE is high [30]. ${\mathrm{DU}}_{l}$ reserves independent frequency resources for each E2E path. Due to the randomness of the wireless channel, whether the UE accesses ${\mathrm{DU}}_{l}$ successfully is stochastic. In Figure 1b, $s_{l}^{1}\left(t\right)$ denotes the service rate provided by ${\mathrm{DU}}_{l}$ at time slot *t*. The service process $\left\{s_{l}^{1}\left(t\right),t\geq 0\right\}$ is assumed to be i.i.d. across slots and can be modeled as

(3) $$\begin{array}{c} s_{l}^{1}\left(t\right)=\left\{\begin{array}{c} C_{l}^{1},ps\\ 0,1-ps \end{array}\right.\left[\mathrm{packets}/\mathrm{slot}\right], \end{array}$$

where $C_{l}^{1}=\left\lfloor \frac{R_{l}^{1}\alpha }{\beta }\right\rfloor$ is the service rate provided by ${\mathrm{DU}}_{l}$ to the UE if it accesses successfully at time slot *t*. α s/slot is the duration of a time slot and β bits/packet is the length of a data packet. ps is the successful access probability.

In the forwarding nodes and the edge server, the service rate can be transformed as $C_{l}^{h}=\left\lfloor \frac{R_{l}^{h}\left(t\right)\alpha }{\beta }\right\rfloor ,h\in H_{l}\setminus \left\{1\right\},l\in L$ packets/slot.

The transmission schemes of the wireless access node and forwarding nodes are different for the UE. Thus, the service processes $\left\{s_{l}^{1}\left(t\right),t\geq 0\right\}$ and $\left\{s_{l}^{h}\left(t\right),t\geq 0\right\},h\in H_{l}\setminus \left\{1\right\}$ are heterogeneous. The arrival process is assumed to be statistically independent of the service processes at all nodes. Meanwhile, the service processes at different nodes along an E2E path are assumed to be mutually independent.

The CU is assumed to have sufficient processing capacity and is not modeled as a queuing node [31,32]. This is because the CU is typically deployed in a centralized data center with abundant computing resources, and its processing delay is small and stable compared with the random access delay at the DU and the forwarding delay at the forwarding nodes. The E2E delay considered in this paper consists of the random access delay at the DU and the queuing/forwarding delay at the forwarding nodes. Propagation delay and processing delay are not separately modeled, since they are negligible compared with the queuing and access delays.

The accumulated arrival process and the accumulated service processes in [m,n] are $A_{l}\left(m,n\right)=\sum_{t=m+1}^{n}a_{l}\left(t\right)$ and $S_{l}^{h}\left(m,n\right)=\sum_{t=m+1}^{n}s_{l}^{h}\left(t\right)$, respectively. Based on the SNC theory, for the E2E path *l*, the departure process of ectype traffic $\left\{a_{l}\left(t\right),t\geq 0\right\}$ at the last node $H_{l}$, $D_{l}\left(0,t\right)$ is derived as

(4) $$\begin{array}{cc} D_{l}\left(0,t\right) & \geq A_{l}⊗S_{l}^{1}⊗S_{l}^{2}⊗\cdots ⊗S_{l}^{H_{l}}\left(0,t\right)\\ \; & =\underset{0\leq m_{1}\leq m_{2}\leq \cdots \leq m_{H_{l}}\leq t}{\inf}\left\{A_{l}\left(0,m_{1}\right)+S_{l}^{1}\left(m_{1},m_{2}\right)+S_{l}^{2}\left(m_{2},m_{3}\right)+\cdots +S_{l}^{H_{l}}\left(m_{H_{l}},t\right)\right\}. \end{array}$$

The E2E delay of $\left\{a_{l}\left(t\right),t\geq 0\right\}$ at slot *t*, $W_{l}\left(t\right)$, is defined as

(5) $$\begin{array}{cc} W_{l}\left(t\right) & \leq \inf\{w\geq 0:A_{l}\left(0,t-w\right)\leq D_{l}\left(0,t\right)\}. \end{array}$$

The data traffic from the UE requires statistical delay QoS provisioning. That is, the probability of the E2E delay exceeding a threshold $W_{\max}$ should be limited below a reliability requirement ζ. In this paper, we focus on exploring an upper bound of the delay violation probability of each E2E path, which is given as $F_{l}(a_{l}\left(t\right),S_{l}\left(t\right),|H_{l}\left|,W_{\max}\right)$, where $S_{l}\left(t\right)={\left[s_{l}^{h}\left(t\right)\right]}_{h\in H_{l}}$. $P\left\{W_{l}\left(t\right)\geq W_{\max}\right\}\leq F_{l}(a_{l}\left(t\right),S_{l}\left(t\right),|H_{l}\left|,W_{\max}\right)$ holds. $F_{l}(a_{l}\left(t\right),S_{l}\left(t\right),|H_{l}\left|,W_{\max}\right)$ is a function of the arrival process, the service process of each node, the hop-count and the threshold.

For the reliability analysis of the parallel transmission system, the E2E paths are modeled as independent reliability branches. After packet duplication, each copy is transmitted through a separately provisioned E2E path with logically isolated network resources. In URLLC-oriented multi-connectivity, duplicated packets can be transmitted over independent links to improve reliability [29]. When the parallel transmission paths are independent and uncorrelated, the system reliability can be expressed in a product form [33]. Following this reliability formulation, the E2E delay violation events of different paths are assumed to be statistically independent. Leveraging the reliability theory, the reliability with regard to E2E delay of the parallel system constructed for the UE, *R*, can be modeled as

(6) $$\begin{array}{c} \begin{array}{c} R=1-\prod_{l\in L}F_{l}(a_{l}\left(t\right),S_{l}\left(t\right),|H_{l}\left|,W_{\max}\right). \end{array} \end{array}$$

The reliability of the parallel system should meet the requirement, i.e., R≥1−ζ. In this paper, the reliability requirement is provisioned by decomposing ζ to each E2E path. According to the service schemes of involved nodes and the number of hops, the maximum reliability that E2E path *l* can carry could be determined by considering the statistical features of the arrival traffic.

## 4. Martingale-Based Upper Bound of E2E Delay Violation Probability

In this section, an analysis framework of the upper bound of E2E delay violation probability, $F_{l}(a_{l}\left(t\right),S_{l}\left(t\right),|H_{l}\left|,W_{\max}\right)$, is constructed based on martingale theory. The martingale processes related to the arrival process and the service processes are defined by leveraging the effective bandwidth and the effective capacity theories. Relying on the Doob maximum inequality of martingales, a close form of $F_{l}(a_{l}\left(t\right),S_{l}\left(t\right),|H_{l}\left|,W_{\max}\right)$ is derived. Throughout this paper, the proposed bound is referred to as a tight upper bound in the following sense. Among upper bounds derived under comparable system assumptions, it yields the smallest gap to the actual delay violation probability, i.e., it is the closest to the simulation results over the considered parameter range. It is not claimed to be an exact characterization of the delay violation probability, since the derivation involves bounding steps such as supremum decomposition and Doob-type inequalities, which are inherently conservative.

A. Martingales related to arrival and service processes

Firstly, we introduce the definition of martingales.

**Definition** **1** (martingales)**.** *Let $\left(\Omega ,F_{\infty },P\right)$ be a probability space and ${\left\{F_{t}\right\}}_{t\geq 0}$ be a filtration, i.e., a non-decreasing sequence of σ-fields of $F_{\infty }$, $F_{t}\subset F_{t+1},t\geq 0$. A random sequence {X(t),t≥0} is adapted to ${\left\{F_{t}\right\}}_{t\geq 0}$. That is, X(t) is measurable with respect to $F_{t},\forall t\geq 0$. If*

*(1)*  *E[|X(t)|]<∞,∀t≥0,*
*(2)*  *$\;E[X\left(t+1\right)|F_{t}]=X\left(t\right),\forall t\geq 0$,*

*hold, then {X(t),t≥0} is a martingale sequence.*

Further, if $\;E[X\left(t+1\right)|F_{t}]\geq X\left(t\right)$, then {X(t),t≥0} is a submartingale, otherwise is a supermartingale.

We consider the E2E traffic possesses i.i.d. arrival features across slots. Then, the martingale related to arrival is constructed in Definition 2.

**Definition** **2** (martingale related to arrival)**.** *The arrival process of UE at E2E path l,l∈L is $\left\{a_{l}\left(t\right),t\geq 0\right\}$ with i.i.d. statistical features, which is measurable for $F_{t}$. The accumulated arrival process is $\left\{A_{l}\left(m,t\right),t\geq m\geq 0\right\}$. The effective bandwidth of $\left\{a_{l}\left(t\right),t\geq 0\right\}$ can be derived as $EB^{l}\left(\theta \right)=\frac{\ln E[e^{\theta a_{l}\left(t\right)}]}{\theta },\forall \theta >0$. A bias scale function of arrival is defined as $\Psi_{a}^{l}\left(m,t\right)=A_{l}\left(m,t\right)-\left(t-m\right)EB^{l}\left(\theta \right)$. The random process $\left\{M_{a}^{l}\left(m,t\right),t\geq m\geq 0\right\}$ is defined as $M_{a}^{l}\left(m,t\right)=e^{\theta \Psi_{a}^{l}\left(m,t\right)}$.*

**Proposition 1.** 
*The random process $\left\{M_{a}^{l}\left(m,t\right),t\geq m\geq 0\right\}$, where $M_{a}^{l}\left(m,t\right)=e^{\theta \Psi_{a}^{l}\left(m,t\right)}$, is a martingale relative to ${\left\{F_{t}\right\}}_{t\geq 0}$.*

**Proof.**  According to $EB^{l}\left(\theta \right)=\frac{\ln E[e^{\theta a_{l}\left(t\right)}]}{\theta }<\infty ,\forall \theta >0$, $E[|M_{a}^{l}\left(m,t\right)|]<\infty ,\forall t\geq 0$, i.e., (1) in Definition 1 can hold. For (2), it can be derived that

(7) $$\begin{array}{cc} E\left[M_{a}^{l}\left(m,t+1\right)|F_{t}\right]\; & =E\left[e^{\theta \Psi_{a}^{l}\left(m,t+1\right)}|F_{t}\right]\\ \; & =E\left[e^{\theta \left[A_{l}\left(m,t+1\right)-\left(t+1-m\right)\frac{\ln E[e^{\theta a_{l}\left(t\right)}]}{\theta }\right]}|F_{t}\right]\\ \; & \overset{\left(a\right)}{=}e^{\theta \left[A_{l}\left(m,t\right)-\left(t+1-m\right)\frac{\ln E[e^{\theta a_{l}\left(t\right)}]}{\theta }\right]}E\left[e^{\theta a_{l}\left(t+1\right)}\right]\\ \; & \overset{\left(b\right)}{=}e^{\theta \left[A_{l}\left(m,t\right)-\left(t+1-m\right)\frac{\ln E[e^{\theta a_{l}\left(t\right)}]}{\theta }\right]}E\left[e^{\theta a_{l}\left(t\right)}\right]\\ \; & =M_{a}^{l}\left(m,t\right). \end{array}$$

(a) is from the i.i.d. feature of arrival process $\left\{a_{l}\left(t\right),t\geq 0\right\}$. (b) holds due to the moment generation function of $\left\{a_{l}\left(t\right),t\geq 0\right\}$. That is, $E\left[e^{\theta a_{l}\left(t+1\right)}\right]=E\left[e^{\theta a_{l}\left(t\right)}\right]$. The proof is completed.    □

The effective bandwidth can be regarded as the minimum service capacity the arrival traffic demands to meet the QoS requirements by considering the statistical characteristics of the traffic [4]. The bias scale function $\Psi_{a}^{l}\left(m,t\right)$ is introduced to measure the difference between the accumulated arrival load and the minimum service that should be provided for $\left\{a_{l}\left(t\right),t\geq 0\right\}$. The e-exponential function can facilitate to amplify the bias. The martingale process $\left\{M_{a}^{l}\left(m,t\right),t\geq m\geq 0\right\}$ can expose the fluctuation of the arrival process and reveal the influence of randomness of traffic on the system delay.

Corresponding to the martingale related to arrival, the martingale processes related to the service process of each node can be constructed in Definition 3.

**Definition 3** 
(martingale related to service). *The service process of node $h,h\in H_{l},l\in L$ is $\left\{s_{l}^{h}\left(t\right),t\geq 0\right\}$ with i.i.d. characteristics, which is measurable for $G_{t}$. The accumulated service process is $\left\{S_{l}^{h}\left(m,t\right),t\geq m\geq 0\right\}$. The effective capacity of $\left\{s_{l}^{h}\left(t\right),t\geq 0\right\}$ can be derived as $EC^{l,h}\left(\theta \right)=-\frac{\ln E\left[e^{-\theta s_{l}^{h}\left(t\right)}\right]}{\theta },\forall \theta >0$. A bias scale function of service is defined as $\Psi_{s}^{l,h}\left(m,t\right)=\left(t-m\right)EC^{l,h}\left(\theta \right)-S_{l}^{h}\left(m,t\right)$. The random process $\left\{M_{s}^{l,h}\left(m,t\right),t\geq m\geq 0\right\}$ is defined as $M_{s}^{l,h}\left(m,t\right)=e^{\theta \Psi_{s}^{l,h}\left(m,t\right)}$.*

**Proposition 2.** 
*The random process $\left\{M_{s}^{l,h}\left(m,t\right),t\geq m\geq 0\right\}$, where $M_{s}^{l,h}\left(m,t\right)=e^{\theta \Psi_{s}^{l,h}\left(m,t\right)}$, is a martingale relative to ${\left\{G_{t}\right\}}_{t\geq 0}$.*

**Proof.**  It is obvious that $E[|M_{s}^{l,h}\left(m,t\right)|]<\infty ,\forall t\geq 0$ holds because of $EC^{l,h}\left(\theta \right)=-\frac{\ln E\left[e^{-\theta s_{l}^{h}\left(t\right)}\right]}{\theta }<\infty ,\forall \theta >0$. For formula (2) in Definition 1, we can write that

(8) $$\begin{array}{cc} E\left[M_{s}^{l,h}\left(m,t+1\right)|G_{t}\right]\; & =E\left[e^{\theta \Psi_{s}^{l,h}\left(m,t+1\right)}|G_{t}\right]\\ \; & =E\left[e^{\theta \left[-\left(t+1-m\right)\frac{\ln E[e^{-\theta s_{l}^{h}\left(t\right)}]}{\theta }-S_{l}^{h}\left(m,t+1\right)\right]}|G_{t}\right]\\ \; & \overset{\left(a\right)}{=}e^{\theta \left[-\left(t+1-m\right)\frac{\ln E[e^{-\theta s_{l}^{h}\left(t\right)}]}{\theta }-S_{l}^{h}\left(m,t\right)\right]}E\left[e^{-\theta s_{l}^{h}\left(t+1\right)}\right]\\ \; & \overset{\left(b\right)}{=}e^{\theta \left[-\left(t+1-m\right)\frac{\ln E[e^{-\theta s_{l}^{h}\left(t\right)}]}{\theta }-S_{l}^{h}\left(m,t\right)\right]}E\left[e^{-\theta s_{l}^{h}\left(t\right)}\right]\\ \; & =M_{s}^{l,h}\left(m,t\right). \end{array}$$

(a) holds relying on the i.i.d. features of $\left\{s_{l}^{h}\left(t\right),t\geq 0\right\}$. According to the property of the moment generation function, $E\left[e^{-\theta s_{l}^{h}\left(t+1\right)}\right]=E\left[e^{-\theta s_{l}^{h}\left(t\right)}\right]$, which supports (b). The proof is completed.    □

Based on the effective capacity theory, $EC^{l,h}\left(\theta \right)$ of $\left\{s_{l}^{h}\left(t\right),t\geq 0\right\}$ can measure the maximum arrival load that the node can carry under the QoS constraints by considering the service scheme of it [7]. Thus, we define the bias scale function of service process $\left\{s_{l}^{h}\left(t\right),t\geq 0\right\}$, $\Psi_{s}^{l,h}\left(m,t\right)$ to depict the difference between the maximum accumulated load and the actual accumulated service. Minor changes of the bias scale function can be amplified through the e-exponential transformation, which contributes to reflect the randomness of the service processes.

Based on the definition of martingales related to arrival and service, we proposed a martingale parameter to model the service ability of the E2E path *l* for a special arrival process, which is defined as service index $\theta_{l}^{*}$. The E2E path *l* is modeled as a tandem queuing system. The overall service ability is determined by all involved nodes.

**Definition 4** 
(service index). *For the arrival process $\left\{a_{l}\left(t\right),t\geq 0\right\}$, the service process of node $h,h\in H_{l},l\in L$ providing for it is $\left\{s_{l}^{h}\left(t\right),t\geq 0\right\}$. There exists a parameter $\theta_{l,h}^{*}\geq 0$ meeting*

(9) $$\begin{array}{c} \theta_{l,h}^{*}=\sup\left\{\theta >0:EB^{l}\left(\theta \right)\leq EC^{l,h}\left(\theta \right)\right\}, \end{array}$$

*which measures the service ability of node h carrying $\left\{a_{l}\left(t\right),t\geq 0\right\}$. Then, the service index $\theta_{l}^{*}$ is defined as*

(10) $$\begin{array}{c} \begin{array}{c} \theta_{l}^{*}=\min_{h\in H_{l}}\sup\left\{\theta >0:EB^{l}\left(\theta \right)\leq EC^{l,h}\left(\theta \right)\right\}, \end{array} \end{array}$$

*which indicates that $\theta_{l}^{*}=\min_{h\in H_{l}}\theta_{l,h}^{*}$, $\theta_{l}^{*}\geq 0$.*

When $\theta =\theta_{l}^{*}$, $\left\{M_{a}^{l}\left(m,t\right),t\geq m\geq 0\right\}$ and $\left\{M_{s}^{l,h}\left(m,t\right),t\geq m\geq 0\right\}$ are still the martingale processes. To achieve the analysis of the E2E delay violation probability, the statistical characteristics of the bias scale functions should be explored. For the functions $\Psi_{a}^{l}\left(m,t\right)$ and $\Psi_{s}^{l,h}\left(m,t\right)$, we can derive the related probability distributions based on the Doob maximum inequality, as presented in Lemma 1.

**Lemma 1.** 
*Within different observation time ranges, the probability distributions of $\Psi_{a}^{l}\left(m,t\right)$ and $\Psi_{s}^{l,h}\left(m,t\right)$ satisfy*

(11) $$\begin{array}{cc} P\{\underset{0\leq m\leq t}{\sup}\theta \Psi_{a}^{l}\left(m,t\right)\geq x\} & \leq e^{-x},\\ P\left\{\underset{0\leq m\leq n\leq t}{\sup}\theta \Psi_{s}^{l,h}\left(m,n\right)\geq x\right\} & \leq e\cdot e^{-x},\\ P\left\{\underset{0\leq m\leq t}{\sup}\theta \Psi_{s}^{l,h}\left(m,t\right)\geq x\right\} & \leq e^{-x}. \end{array}$$

**Proof.**  According to Doob maximum inequality of martingale processes, if {X(t),t≥0} is a submartingale, $P\{\underset{0\leq m\leq t}{\sup}X\left(m\right)\geq x\}\leq E[X\left(t\right)]x^{-1}$, where *x* is a constant. If {X(t),t≥0} is a martingale, $P\{\underset{0\leq m\leq t}{\sup}\left|X\left(m\right)\right|\geq x\}\leq E[|X\left(t\right)|]x^{-1}$. Then, for $\underset{0\leq m\leq t}{\sup}\theta \Psi_{a}^{l}\left(m,t\right)$ and $\underset{0\leq m\leq t}{\sup}\theta \Psi_{s}^{l,h}\left(m,t\right)$, we can derive that

(12) $$\begin{array}{cc} P\left\{\underset{0\leq m\leq t}{\sup}\theta \Psi_{a}^{l}\left(m,t\right)\geq x\right\}\; & =P\left\{\underset{0\leq m\leq t}{\sup}M_{a}^{l}\left(m,t\right)\geq e^{x}\right\}\\ \; & \leq E\left[M_{a}^{l}\left(m,t\right)\right]e^{-x}=e^{-x}. \end{array}$$

The last step relies on the features of martingales. And $P\left\{\underset{0\leq m\leq t}{\sup}\theta \Psi_{s}^{l,h}\left(m,t\right)\geq x\right\}\leq e^{-x}$ follows the same line.

For $\underset{0\leq m\leq n\leq t}{\sup}\theta \Psi_{s}^{l,h}\left(m,n\right)$, we tackle it as (13):

(13) $$\begin{array}{cc} P\left\{\underset{0\leq m\leq n\leq t}{\sup}\theta \Psi_{s}^{l,h}\left(m,n\right)\geq x\right\}\; & =P\left\{\underset{0\leq n\leq t}{\sup}\left[\underset{0\leq m\leq n}{\sup}M_{s}^{l,h}\left(m,n\right)\right]\geq e^{x}\right\}. \end{array}$$

It is defined that $\hat{M_{s}^{l,h}}\left(m,n\right)=\underset{0\leq m\leq n}{\sup}M_{s}^{l,h}\left(m,n\right)$. Based on Definition 1, we can derive that

(14) $$\begin{array}{cc} E\left[\hat{M_{s}^{l,h}}\left(m,t+1\right)|G_{t}\right]\; & \overset{\left(a\right)}{\geq }E\left\{\underset{0\leq m\leq t}{\sup}\left\{e^{\theta \Psi_{s}^{l,h}\left(m,t+1\right)}|G_{t}\right\}\right\}\\ \; & =E\left\{\underset{0\leq m\leq t}{\sup}\left\{e^{\theta \left[-\left(t+1-m\right)\frac{\ln E[e^{-\theta s_{l}^{h}\left(t\right)}]}{\theta }-S_{l}^{h}\left(m,t+1\right)\right]}|G_{t}\right\}\right\}\\ \; & =\hat{M_{s}^{l,h}}\left(m,t\right)\;E\left[e^{\theta \left(-\frac{\ln E[e^{-\theta s_{l}^{h}\left(t\right)}]}{\theta }-s_{l}^{h}\left(t+1\right)\right)}\right]\\ \; & =\hat{M_{s}^{l,h}}\left(m,t\right). \end{array}$$

(a) holds from the reduction of supremum from *t* + 1 to *t* and the independent identical distributed feature. It proves that $\hat{M_{s}^{l,h}}\left(t\right)$ is a submartingale process. Based on this conclusion, we can obtain (15) for (13).

(15) $$\begin{array}{cc} P\left\{\underset{0\leq n\leq t}{\sup}\left[\underset{0\leq m\leq n}{\sup}M_{s}^{l,h}\left(m,n\right)\right]\geq e^{x}\right\}\; & =P\left\{\underset{0\leq n\leq t}{\sup}\left[\hat{M_{s}^{l,h}}\left(m,n\right)\right]\geq e^{x}\right\}\\ \; & \leq E\left[\hat{M_{s}^{l,h}}\left(m,t\right)\right]e^{-x}\\ \; & \leq e\cdot e^{-x}. \end{array}$$

The last step relies on Holder inequality and L’Hospital’s rule. The proof is completed.    □

Leveraging the definitions of martingales related to arrival and service, the introduction of service index, as well as Remark 1, the upper bound of the E2E delay violation probability can be derived.

B. The upper bound of the E2E delay violation probability

The martingale-based theoretical result of the E2E delay violation probability is shown in Theorem 1.

**Theorem 1.** 
*The arrival process $\left\{a_{l}\left(t\right),t\geq 0\right\}$ possesses i.i.d. features, and the martingale related to it is shown in Definition 2. The E2E delay threshold is $W_{\max}$. In the E2E path l,l∈L constructed for $\left\{a_{l}\left(t\right),t\geq 0\right\}$, the service process of involved node $h,h\in H_{l}$ is $\left\{s_{l}^{h}\left(t\right),t\geq 0\right\}$ and the corresponding martingale related to it is in Definition 3. The arrival process is independent of the service processes, which are mutually independent across different nodes. The hop counts is $|H_{l}|$. For this tandem queuing system, the service index is introduced in Definition 4. For $\forall W_{\max}>0$, the upper bound of the E2E delay violation probability, $F_{l}(a_{l}\left(t\right),S_{l}\left(t\right),|H_{l}\left|,W_{\max}\right)$, can be given as*

(16) $$\begin{array}{cc} P\{W_{l}\left(t\right)\geq W_{\max}\}\; & \leq F_{l}(a_{l}\left(t\right),S_{l}\left(t\right),|H_{l}\left|,W_{\max}\right)\\ \; & =e^{\left(\right|H_{l}\left|-1\right)-\theta_{l}^{*}EB^{l}\left(\theta_{l}^{*}\right)W_{\max}}\sum_{h=0}^{|H_{l}|}\frac{{\left[\theta_{l}^{*}EB^{l}\left(\theta_{l}^{*}\right)W_{\max}-\left(|H_{l}|-1\right)\right]}^{h}}{h!}. \end{array}$$

**Proof.**  According to the definition of E2E delay (5), it can be written that

(17) $$\begin{array}{cc} P\{W_{l}\left(t\right)\geq W_{\max}\}\; & \leq P\left\{\underset{0\leq m_{1}\leq \cdots \leq m_{H_{l}}\leq t}{\sup}\left[A_{l}\left(m_{1},t-W_{\max}\right)-\sum_{h=1}^{|H_{l}|-1}S_{l}^{h}\left(m_{h},m_{h+1}\right)-S_{l}^{H_{l}}\left(m_{H_{l}},t\right)\right]\geq 0\right\}. \end{array}$$

$\theta_{l,h}^{*}$ is introduced to tackle (17). When it is set as $\theta_{l,h}^{*}=\sup\left\{\theta >0:EB^{l}\left(\theta \right)=EC^{l,h}\left(\theta \right)\right\},$ $h\in H_{l}$, Formula (18) (a) holds. (18) (b) can be derived relying on the definition of service index $\theta_{l}^{*}=\min_{h\in H_{l}}\theta_{l,h}^{*}$. (18) (c) is from $\underset{0\leq m\leq t}{\sup}\left\{X\left(m\right)+Y\left(m\right)\right\}\leq \underset{0\leq m\leq t}{\sup}\left\{X\left(m\right)\right\}+\underset{0\leq m\leq t}{\sup}\left\{Y\left(m\right)\right\}$. And (18) (d) holds because the definition of bias scale functions of arrival and service, i.e., $\Psi_{a}^{l}\left(m,t\right)$ and $\Psi_{s}^{l,h}\left(m,t\right)$.

(18) $$\begin{array}{cc} \; & P\left\{\underset{0\leq m_{1}\leq \cdots \leq m_{H_{l}}\leq t}{\sup}\left[A_{l}\left(m_{1},t-W_{\max}\right)-\sum_{h=1}^{|H_{l}|-1}S_{l}^{h}\left(m_{h},m_{h+1}\right)-S_{l}^{H_{l}}\left(m_{H_{l}},t\right)\right]\geq 0\right\}\overset{\left(a\right)}{=}\\ \; & P\left\{\underset{0\leq m_{1}\leq \cdots \leq m_{H_{l}}\leq t}{\sup}\left[A_{l}\left(m_{1},t-W_{\max}\right)-\sum_{h=1}^{|H_{l}|-1}S_{l}^{h}\left(m_{h},m_{h+1}\right)-S_{l}^{H_{l}}\left(m_{H_{l}},t\right)\right.\right.\\ \; & \;\;\left.\left.-\sum_{h=1}^{|H_{l}|-1}EB^{l}\left(\theta_{l,h}^{*}\right)\left(m_{h+1}-m_{h}\right)-EB^{l}\left(\theta_{l,H_{l}}^{*}\right)\left(t-m_{H_{l}}\right)\right.\right.\\ \; & \;\;\left.\left.+\sum_{h=1}^{|H_{l}|-1}EC^{l,h}\left(\theta_{l,h}^{*}\right)\left(m_{h+1}-m_{h}\right)+EC^{l,H_{l}}\left(\theta_{l,H_{l}}^{*}\right)\left(t-m_{H_{l}}\right)\right]\geq 0\right\}\overset{\left(b\right)}{\leq }\\ \; & P\left\{\underset{0\leq m_{1}\leq \cdots \leq m_{H_{l}}\leq t}{\sup}\theta_{l}^{*}\left[A_{l}\left(m_{1},t-W_{\max}\right)-\sum_{h=1}^{|H_{l}|-1}S_{l}^{h}\left(m_{h},m_{h+1}\right)-S_{l}^{H_{l}}\left(m_{H_{l}},t\right)\right.\right.\\ \; & \;\;\left.\left.-EB^{l}\left(\theta_{l}^{*}\right)\left(t-W_{\max}-m_{1}\right)+\sum_{h=1}^{|H_{l}|-1}EC^{l,h}\left(\theta_{l}^{*}\right)\left(m_{h+1}-m_{h}\right)\right.\right.\\ \; & \;\;\left.\left.+EC^{l,H_{l}}\left(\theta_{l}^{*}\right)\left(t-m_{H_{l}}\right)\right]\geq \theta_{l}^{*}EB^{l}\left(\theta_{l}^{*}\right)W_{\max}\right\}\overset{\left(c\right)}{\leq }\\ \; & P\left\{\underset{0\leq m_{1}\leq t}{\sup}\theta_{l}^{*}\left[A_{l}\left(m_{1},t-W_{\max}\right)-EB^{l}\left(\theta_{l}^{*}\right)\left(t-W_{\max}-m_{1}\right)\right]\right.\\ \; & \;\;\left.+\sum_{h=1}^{|H_{l}|-1}\underset{0\leq m_{h}\leq m_{h+1}\leq t}{\sup}\theta_{l}^{*}\left[EC^{l,h}\left(\theta_{l}^{*}\right)\left(m_{h+1}-m_{h}\right)-S_{l}^{h}\left(m_{h},m_{h+1}\right)\right]\right.\\ \; & \;\;\left.+\underset{0\leq m_{H_{l}}\leq t}{\sup}\theta_{l}^{*}\left[EC^{l,H_{l}}\left(\theta_{l}^{*}\right)\left(t-m_{H_{l}}\right)-S_{l}^{H_{l}}\left(m_{H_{l}},t\right)\right]\geq \theta_{l}^{*}EB^{l}\left(\theta_{l}^{*}\right)W_{\max}\right\}\overset{\left(d\right)}{=}\\ \; & P\left\{\underset{0\leq m_{1}\leq t}{\sup}\theta_{l}^{*}\left[\Psi_{a}^{l}\left(m_{1},t-W_{\max}\right)\right]+\sum_{h=1}^{|H_{l}|-1}\underset{0\leq m_{h}\leq m_{h+1}\leq t}{\sup}\theta_{l}^{*}\left[\Psi_{s}^{l,h}\left(m_{h},m_{h+1}\right)\right]\right.\\ \; & \;\;\left.+\underset{0\leq m_{H_{l}}\leq t}{\sup}\theta_{l}^{*}\left[\Psi_{s}^{l,H_{l}}\left(m_{H_{l}},t\right)\right]\geq \theta_{l}^{*}EB^{l}\left(\theta_{l}^{*}\right)W_{\max}\right\}. \end{array}$$

The E2E delay violation probability is related to the probability distributions of $\Psi_{a}^{l}\left(m,t\right)$ and $\Psi_{s}^{l,h}\left(m,t\right),h\in H_{l}$. Leveraging Lemma 1, we can analyze that the formulas in (19) hold when $x=\theta_{l}^{*}EB^{l}\left(\theta_{l}^{*}\right)W_{\max}$.

(19) $$\begin{array}{cc} \; & P\left\{\underset{0\leq m_{1}\leq t}{\sup}\theta_{l}^{*}\Psi_{a}^{l}\left(m_{1},t-W_{\max}\right)\geq x\right\}\leq e^{-x},\\ \; & P\left\{\underset{0\leq m_{h}\leq m_{h+1}\leq t}{\sup}\theta_{l}^{*}\Psi_{s}^{l,h}\left(m_{h},m_{h+1}\right)\geq x\right\}\leq e\cdot e^{-x},\;h\in \left[1,|H_{l}|-1\right]\\ \; & P\left\{\underset{0\leq m_{H_{l}}\leq t}{\sup}\theta_{l}^{*}\Psi_{s}^{l,H_{l}}\left(m_{H_{l}},t\right)\geq x\right\}\leq e^{-x}. \end{array}$$

We denote $\underset{0\leq m_{1}\leq t}{\sup}\theta_{l}^{*}\Psi_{a}^{l}\left(m_{1},t-W_{\max}\right)$, $\underset{0\leq m_{h}\leq m_{h+1}\leq t}{\sup}\theta_{l}^{*}\Psi_{s}^{l,h}\left(m_{h},m_{h+1}\right),h\in \left[1,|H_{l}|-1\right]$ and $\underset{0\leq m_{H_{l}}\leq t}{\sup}\theta_{l}^{*}\Psi_{s}^{l,H_{l}}\left(m_{H_{l}},t\right)$ as *U*, $V_{h},h\in \left[1,|H_{l}|-1\right]$ and $V_{H_{l}}$, respectively. *U*, $V_{h},h\in \left[1,|H_{l}|-1\right]$ and $V_{H_{l}}$ are all random variables and can be regarded as obeying independent exponential distributions related to *x*. The moment generating functions are derived as $M_{U}\left(k\right),\;M_{V_{h}}\left(k\right),\;M_{V_{H_{l}}}\left(k\right)$, respectively:

(20) $$\begin{array}{cc} M_{U}\left(k\right) & =\frac{1}{1-k},\\ M_{V_{h}}\left(k\right) & =\frac{e^{k}}{1-k},\\ M_{V_{H_{l}}}\left(k\right) & =\frac{1}{1-k}. \end{array}$$

Thus, relying on the independent features, the moment generating function of $U+\sum_{h=1}^{|H_{l}|-1}V_{h}+V_{H_{u,l}}$ can be given as

(21) $$M\left(k\right)={\left(\frac{1}{1-k}\right)}^{|H_{l}|+1}e^{k(|H_{l}|-1)}.$$

Formula (21), we can judge that random variable $U+\sum_{h=1}^{|H_{l}|-1}V_{h}+V_{H_{l}}$ follows a gamma distribution, where the parameters are $|H_{l}|+1$ and $\left[\theta_{l}^{*}EB^{l}\left(\theta_{l}^{*}\right)W_{\max}-\left(|H_{l}|-1\right)\right]$. Based on [3], we can write that

(22) $$\begin{array}{cc} P\{W_{l}\left(t\right)\geq W_{\max}\}\; & \leq P\left\{U+\sum_{h=1}^{|H_{l}|-1}V_{h}+V_{H_{l}}\geq x\right\}\\ \; & =e^{\left(\right|H_{l}\left|-1\right)-\theta_{l}^{*}EB^{l}\left(\theta_{l}^{*}\right)W_{\max}}\sum_{h=0}^{|H_{l}|}\frac{{\left[\theta_{l}^{*}EB^{l}\left(\theta_{l}^{*}\right)W_{\max}-\left(|H_{l}|-1\right)\right]}^{h}}{h!}. \end{array}$$

The proof is completed.    □

## 5. The Bandwidth Demands Capture and Reliability Decomposition Scheme

In this paper, the E2E delay QoS requirements of the target traffic are provisioned by a parallel transmission system consisting of multiple independent E2E paths. According to (6), the system reliability should satisfy R≥1−ζ under the E2E delay threshold $W_{\max}$. The reliability requirement should be decomposed to different E2E paths to carry, while the service configurations of involved nodes in each E2E path determine the maximum reliability that can be provided to the traffic. These two issues couple with each other. Thus, in this section, we proposed a bandwidth demands capture and reliability decomposition (BDC-RD) scheme. Relying on the martingale-based upper bound of E2E delay violation probability, the E2E delay reliability requirement is decomposed and the service demands of each node in the parallel system are determined.

Under the E2E delay threshold $W_{\max}$ and reliability requirement ζ constraints, the bandwidth demands that each node should provide are denoted as $C_{l}^{h},h\in H_{l},l\in L$ packets/slot, which forms the bandwidth demands matrix $C={\left[C_{l}^{h}\right]}_{l\in L,h\in H_{l}}$. Specifically, $C_{l}^{1},l\in L$ is the service rate provided by the DU belonging to E2E path *l* if the ectype arrival traffic accesses successfully. $C_{l}^{h},h\in H_{l}\setminus \left\{1\right\},l\in L$ is the service rate that should be offered by the related router node. An optimization problem about E2E delay QoS provisioning can be formulated as follows:

(23) $$\begin{array}{c} \min_{C}\sum_{l\in L}\sum_{h\in H_{l}}C_{l}^{h} \end{array}$$

(24) $$\begin{array}{c} s.t.\;\prod_{l\in L}e^{\left(\right|H_{l}\left|-1\right)-\theta_{l}^{*}EB^{l}\left(\theta_{l}^{*}\right)W_{\max}}\sum_{h=0}^{|H_{l}|}\frac{{\left[\theta_{l}^{*}EB^{l}\left(\theta_{l}^{*}\right)W_{\max}-\left(|H_{l}|-1\right)\right]}^{h}}{h!}\leq \zeta , \end{array}$$

(25) $$\begin{array}{c} C≻0. \end{array}$$

The optimization objective of problem (23) is to minimize the total service rate of the parallel system. The optimization variable is the demanded service rate of each node. (24) indicates that the reliability of the parallel system should meet the reliability requirement ζ leveraging the martingale-based theoretical result. (25) limits the demanded bandwidth of each node. It is unable to obtain the close form of each element in C. In the delay analysis framework based on martingale theory, the service configuration of node *h* indirectly affects delay performance of E2E path *l* by influencing the proposed martingale parameter $\theta_{l,h}^{*}$. And then the service index of an E2E path, $\theta_{l}^{*}$, is involved. Through $\theta_{l}^{*}$, the upper bound of E2E delay violation probability is determined and further the system reliability is depicted. Problem (23) is non-convex and is hard to explore the optimal solution. Based on the PSO search algorithm, we are devoted to capturing a feasible solution of problem (23). Meanwhile, the service index $\theta_{l}^{*}$ offers a descending dimension method for optimization variable. In E2E path *l*, we set $\theta_{l}^{*}$ as $\theta_{l}^{*}=\theta_{l,h}^{*},h\in H_{l}$. It implies that in E2E path *l*, the service ability of each node provided to the arrival process $\left\{a_{l}\left(t\right),t\geq 0\right\}$ is required to be uniform. Based on it, problem (23) can be changed as

(26) $$\begin{array}{c} \min_{C}\sum_{l\in L}\sum_{h\in H_{l}}C_{l}^{h} \end{array}$$

(27) $$\begin{array}{c} s.t.\;\prod_{l\in L}e^{\left(\right|H_{l}\left|-1\right)-\theta_{l}^{*}EB^{l}\left(\theta_{l}^{*}\right)W_{\max}}\sum_{h=0}^{|H_{l}|}\frac{{\left[\theta_{l}^{*}EB^{l}\left(\theta_{l}^{*}\right)W_{\max}-\left(|H_{l}|-1\right)\right]}^{h}}{h!}\leq \zeta , \end{array}$$

(28) $$\begin{array}{c} \theta_{l}^{*}=\theta_{l,h}^{*},h\in H_{l}, \end{array}$$

(29) $$\begin{array}{c} C≻0. \end{array}$$

Leveraging the PSO search algorithm, when the bandwidth demands of any node in E2E path *l* is captured, that of the others can be calculated by solving (26). The pseudocode of the BDC-RD scheme is shown in Algorithm 1.
**Algorithm 1** The BDC-RD scheme.**Input:** The arrival parameters of $\left\{a_{l}\left(t\right),t\geq 0\right\}$. The E2E delay threshold, $W_{\max}$ and reliability requirement, ζ. The successful access probability, ps. The hop counts of each E2E path *l*, $|H_{l}|$. The swarm size: *Z*. The maximum iterations: *N*.**Initialization:** The set of particle swarm, Z. The position of particle *z*, ${\mathrm{POS}}_{z}\left(0\right)$. Personal optimum position, $P_{z}^{\mathrm{best}}\left(0\right)$. The fitness function, $fitnessp_{z}\left(0\right)$. Global optimum position, $G^{\mathrm{best}}\left(0\right)$. The fitness of $G^{\mathrm{best}}\left(0\right)$, fitnessg(0). The velocity of particle *z*, $V_{z}\left(0\right)$.**while ***n* doesn’t reach the maximum iterations *N* **do**    **for** the particle z,z∈Z **do**        **for** *j*th element in ${\mathrm{POS}}_{z}\left(n\right)$ **do**            Update $v_{z}^{j}\left(n+1\right)$ and ${\tilde{C}}_{z}^{j}\left(n+1\right)$.        **end for**        Update ${\mathrm{POS}}_{z}\left(n+1\right)$. According to $|H_{l}|$ and ps, calculate $fitnessp_{z}\left(n+1\right)$.        Comparing $fitnessp_{z}\left(n+1\right)$ and $fitnessp_{z}\left(n\right)$, update $P_{z}^{\mathrm{best}}\left(n+1\right)$.        Comparing $fitnessp_{z}\left(n+1\right)$ and fitnessg(n), update $G^{\mathrm{best}}\left(n+1\right)$.    **end for****end while**$C_{l}^{1}=g_{l}^{\mathrm{best}}\left(N\right),l\in L$.**for** each l∈L **do**    Calculate $\theta_{l,1}^{*}$ according to $C_{l}^{1}$ and ps.    Solve (26).**end for****Output:** C.

In Algorithm 1, the set of particle swarm is denoted as Z. During *n*th iteration, the position of particle z,z∈Z is $POS_{z}\left(n\right)={\left[{\tilde{C}}_{z}^{l}\left(n\right)\right]}_{l\in L}$, where ${\tilde{C}}_{z}^{l}\left(n\right)$ is the service rate of the wireless access network in the E2E path *l* at the current iteration. According to the arrival parameters, the hop counts, and the successful access probability, the fitness function of particle *z* is defined as

(30) $$\begin{array}{c} fitnessp_{z}\left(n\right)=\sum_{l\in L}{\tilde{C}}_{z}^{l}\left(n\right)+G{\left[F_{l}(a_{l}\left(t\right),S_{l}\left(t\right),|H_{l}\left|,W_{\max}\right){|}_{{\left[{\tilde{C}}_{z}^{l}\left(n\right)\right]}_{l\in L}}-\zeta ,0\right]}_{+}, \end{array}$$

where $F_{l}(a_{l}\left(t\right),S_{l}\left(t\right),|H_{l}\left|,W_{\max}\right){|}_{{\left[{\tilde{C}}_{z}^{l}\left(n\right)\right]}_{l\in L}}$ is the value of the upper bound with the service configuration of ${\left[{\tilde{C}}_{z}^{l}\left(n\right)\right]}_{l\in L}$. ${\left[x,0\right]}_{+}=\max\left(x,0\right)$. G≥0 is large enough as a penalty index.

The personal optima position is $P_{z}^{best}\left(n\right)$, which is determined by comparing $fitnessp_{z}\left(n\right)$ and $fitnessp_{z}\left(n-1\right)$. The global optima position is $G^{best}\left(n\right)$ and the corresponding fitness function is fitnessg(n). The velocity of particle *z* is denoted as $V_{z}\left(n\right)={\left[v_{z}^{l}\left(n\right)\right]}_{l\in L}$. $v_{z}^{l}\left(n+1\right)$ is updated as $v_{z}^{l}\left(n+1\right)=wv_{z}^{l}\left(n\right)+\gamma_{1}rand_{1}\left(p_{z,l}^{best}\left(n\right)-{\tilde{C}}_{z}^{l}\left(n\right)\right)+\gamma_{2}rand_{2}\left(g_{l}^{best}\left(n\right)-{\tilde{C}}_{z}^{l}\left(n\right)\right)$, where *w* is inertia weight. $rand_{1}$ and $rand_{2}$ are random numbers distributed in the range [0, 1]. $\gamma_{1}$ and $\gamma_{2}$ are the learning factors. $p_{z,l}^{best}\left(n\right)$ and $g_{l}^{best}\left(n\right)$ are the *l*th elements of $P_{z}^{best}\left(n\right)$ and $G^{best}\left(n\right)$ respectively. And ${\tilde{C}}_{z}^{l}\left(n+1\right)={\tilde{C}}_{z}^{l}\left(n\right)+v_{z}^{l}\left(n+1\right)$.

## 6. The Physical Layer Resource Allocation Algorithm with the Service Rate Demand Constraints

Based on the BDC-RD scheme, the demanded service rates of all the involved nodes can be captured. These are the bandwidth requirements in the view of the MAC layer, which can be used to guide the resource allocation of the physical layer. In the core network, $\frac{\left\lceil C_{l}^{h}\right\rceil \beta }{\alpha },h\in H_{l}\setminus \left\{1\right\},l\in L$ bits/s is the transmission rate that node *h* should provide. In the wireless access network, $C_{l}^{1}$, l∈L, denotes the service rate required upon successful access, which is used to guide transmission power allocation. Accordingly, the power allocation problem at time slot *t* can be formulated as

(31) $$\begin{array}{c} \min_{P(t)}\sum_{l\in L}p_{l}^{1}\left(t\right) \end{array}$$

(32) $$\begin{array}{c} s.t.\;W\log_{2}\left(1+\frac{p_{l}^{1}\left(t\right){h_{l}^{1}}^{2}}{\sigma^{2}}\right)\geq \frac{\left\lceil C_{l}^{1}\right\rceil \beta }{\alpha },\forall l\in L, \end{array}$$

(33) $$\begin{array}{c} \sum_{l\in L}p_{l}^{1}\left(t\right)\leq p^{\max}. \end{array}$$

$P\left(t\right)={\left[p_{l}^{1}\left(t\right)\right]}_{l\in L}$ is the optimal variable matrix. The optimal object of Problem (31) is minimizing the total power consumption of the UE. (32) limits that the transmission rate must meet the service rate demands in terms of bits/s. And (33) is the constraint of the total transmission power. (31) is a convex optimization problem.

According to (31), the Lagrange function can be constructed as

(34) $$\begin{array}{cc} L(P(t),\omega ,\rho )\; & =\sum_{l\in L}p_{l}^{1}\left(t\right)+\sum_{l\in L}\varpi_{l}\left(\frac{\left\lceil C_{l}^{1}\right\rceil \beta }{\alpha }-W\log_{2}\left(1+\frac{p_{l}^{1}\left(t\right){h_{l}^{1}}^{2}}{\sigma^{2}}\right)\right)\\ \; & \;+\rho \left(\sum_{l\in L}p_{l}^{1}\left(t\right)-p^{\max}\right), \end{array}$$

where $\omega ={\left[\varpi_{l}\right]}_{l\in L}$ and ρ are the Lagrange multipliers. Further, the Lagrange dual function can be defined as

(35) $$\begin{array}{c} \hat{L}\left(\omega ,\rho \right)=\min_{P(t)}L\left(P\left(t\right),\omega ,\rho \right). \end{array}$$

Thus, the Lagrange dual problem is formulated as

(36) $$\begin{array}{c} \max_{\omega ,\rho }\min_{P(t)}\sum_{l\in L}p_{l}^{1}\left(t\right)+\sum_{l\in L}\varpi_{l}\left(\frac{\left\lceil C_{l}^{1}\right\rceil \beta }{\alpha }-W\log_{2}\left(1+\frac{p_{l}^{1}\left(t\right){h_{l}^{1}}^{2}}{\sigma^{2}}\right)\right)+\rho \left(\sum_{l\in L}p_{l}^{1}\left(t\right)-p^{\max}\right) \end{array}$$

(37) $$\begin{array}{c} s.t.\;\varpi_{l}\geq 0,\;l\in L, \end{array}$$

(38) $$\begin{array}{c} \rho \geq 0. \end{array}$$

The Lagrange gradient algorithm is leveraged to solve problem (36). In *i*th iteration, the Lagrange multipliers are denoted as $\tilde{\omega }\left(i\right)={\left[{\tilde{\varpi }}_{l}\left(i\right)\right]}_{l\in L}$ and $\tilde{\rho }\left(i\right)$. The allocated transmission power is $\tilde{P}\left(i\right)={\left[{\tilde{p}}_{l}^{1}\left(i\right)\right]}_{l\in L}$, which can be derived as

(39) $$\begin{array}{c} {\tilde{p}}_{l}^{1}\left(i\right)={\left[\frac{{\tilde{\varpi }}_{l}\left(i\right)W}{\ln2(1+\tilde{\rho }\left(i\right))}-\frac{\sigma^{2}}{{h_{l}^{1}}^{2}}\right]}_{+}. \end{array}$$

The Lagrange multipliers are updated as (40) and (41)

(40) $$\begin{array}{c} {\tilde{\varpi }}_{l}\left(i+1\right)={\left[{\tilde{\varpi }}_{l}\left(i\right)+\eta \left(\frac{\left\lceil C_{l}^{1}\right\rceil \beta }{\alpha }-W\log_{2}\left(1+\frac{{\tilde{p}}_{l}^{1}\left(i\right){h_{l}^{1}}^{2}}{\sigma^{2}}\right)\right)\right]}_{+}, \end{array}$$

(41) $$\begin{array}{c} \tilde{\rho }\left(i+1\right)={\left[\tilde{\rho }\left(i\right)+\eta \left(\sum_{l\in L}{\tilde{p}}_{l}^{1}\left(i\right)-p^{\max}\right)\right]}_{+}, \end{array}$$

where η is the learning rate.

## 7. Simulations and Results Analysis

In this section, the effectiveness of the proposed E2E delay QoS guarantee scheme is verified. The performance of the martingale-based theoretical result is analyzed. The simulations are performed to explore the influence factors of E2E delay in MATLAB R2017b.

**Figure 2:**
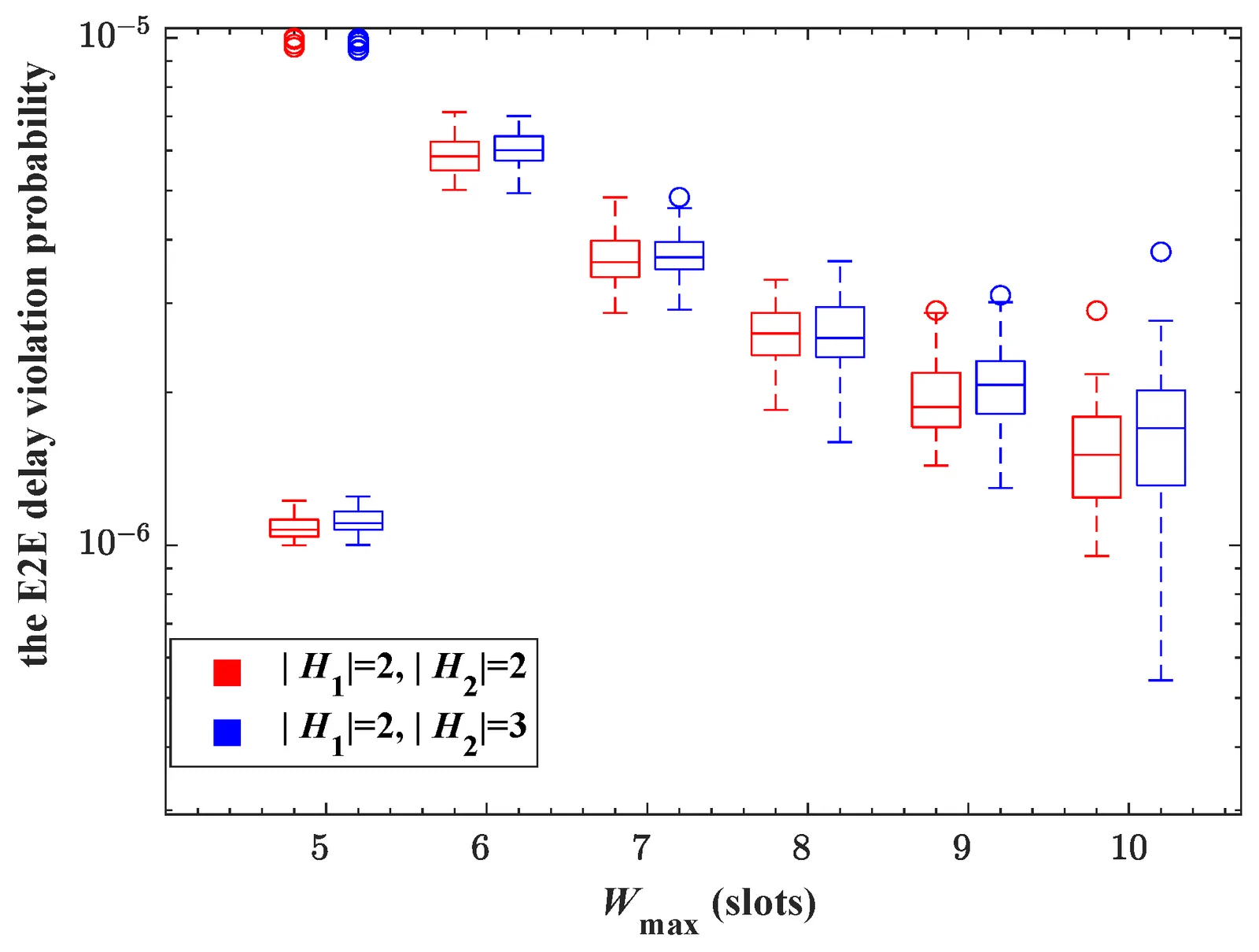
The simulated E2E delay violation probability with two different parallel systems, where λ=5 packets/slot, ps=0.9.

Firstly, we demonstrate the effectiveness and availability of the proposed BDC-RD scheme and physical layer resource allocation algorithm on QoS provisioning. It is considered that the target UE generates the data packets traffic following a Poisson distribution with average arrival rate λ. The E2E delay threshold is $W_{\max}$ and the reliability requirement is ζ. Two E2E paths are constructed for it. That is, |L|=2. To reduce the E2E delay, we assume that the number of hops of each E2E path is determined. The DUs of these two E2E paths offer service processes modeled as (3) independently. The involved forwarding nodes provide constant service rates $C_{l}^{h},h\in H_{l}\setminus \left\{1\right\},l\in L$. Based on Theorem 1, the upper bound of E2E delay violation probability for E2E path *l* can be obtained. The BDC-RD scheme is performed, which captures the demanded service rates $C_{l}^{h},h\in H_{l},l\in L$. Then, the required transmission rate of forwarding nodes are determined. And in the wireless access network, $C_{l}^{1},l\in L$ is used to perform the physical layer resource allocation algorithm. The model of path loss is $\tau \left(d_{l}^{1}\right)=31.5+35\log_{10}\left(d_{l}^{1}\right)$. The log normal shadowing standard deviation is 8 [34]. The transmission power for these two E2E paths are obtained. A tandem queuing system is simulated. The arrival process is copied to two paths for transmission. The E2E delay of $10^{6}$ slots is simulated and recorded for each E2E path. For each time slot, only if the delay of both paths exceeds $W_{\max}$, this slot is labeled as a violated slot. The proportion of violated slots is regarded as the E2E delay violation probability in a simulation. For different delay thresholds, the simulations are repeated for 50 times. The simulated E2E delay violation probabilities are shown as box plots in Figure 2. The common used parameters are summarized in Table 3 unless otherwise specified.

**Figure 3:**
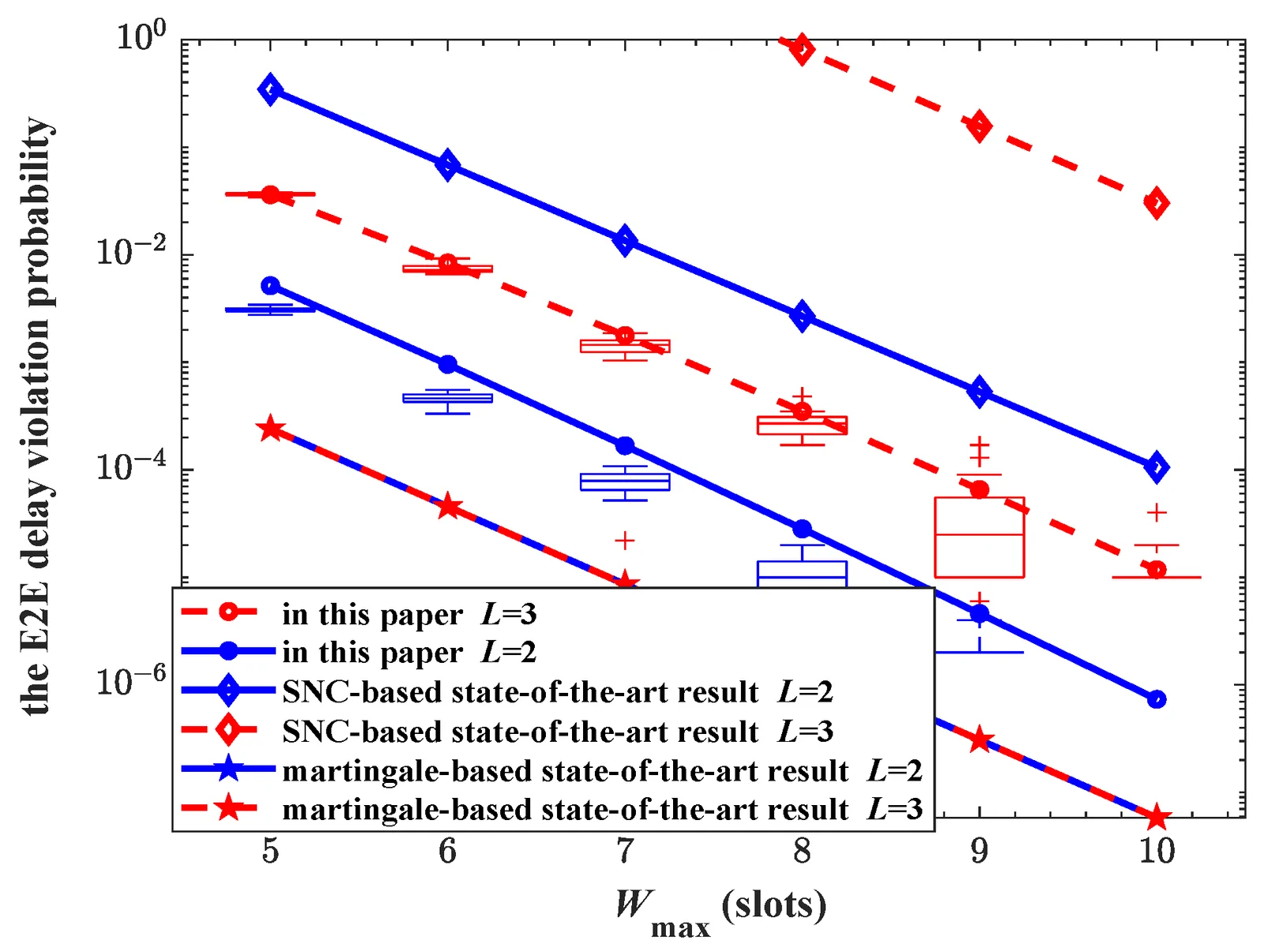
The comparison between the theoretical and simulation results of the E2E delay violation probability, where λ=10 packets/slot, C=12 packets/slot, ps=0.9. L=2 or L=3. Blue and red lines are the theoretical results derived by different methods, and the box plots represent simulation results.

We consider two different parallel systems for the UE, one of which includes two E2E paths with hop counts of $|H_{1}|=2$ and $|H_{2}|=2$, and the others are $|H_{1}|=2$ and $|H_{2}|=3$. It can be seen that with different parallel transmission patterns, the simulated E2E delay violation probabilities are limited below the reliability requirement $\zeta =10^{-5}$. The designed BDC-RD scheme and resource allocation algorithm can achieve the strict E2E delay QoS requirements provisioning flexibly. As the delay threshold relaxes, the proposed bandwidth demands capture scheme can evaluate the service rate requirements of all the involved nodes efficiently so that the reliability requirement ζ can be decomposed reasonably to each E2E paths according to the hop counts and the heterogeneous service schemes. Meanwhile, the resource of the physical layer can be allocated reasonably. The martingale-based E2E delay performance analysis framework supports the effectiveness of the proposed QoS guarantee scheme. The derived upper bound of the E2E delay violation probability characterizes the effects of the stochastic characteristics of arrival traffic, the heterogeneous service processes of DUs and forwarding nodes, and the different hop counts of E2E paths. The E2E delay arises from the interaction of multiple heterogeneous random processes. In this framework, martingales related to the arrival and service processes are introduced to model these heterogeneous random processes in a modular manner. The bias scale functions of arrival and service are defined to characterize the deviations of accumulated arrival and service from their corresponding reference levels, which helps reveal the impacts of their fluctuations on the E2E delay. The Doob maximum inequality is applied to derive an upper bound of the E2E delay violation probability, which provides a theoretical basis for capturing the bandwidth demands. The tightness of the derived upper bound is further evaluated through comparisons with existing theoretical results and simulation results, as shown in Figure 3.

In Figure 3, the martingale-based upper bound of the E2E delay violation probability is compared with the state-of-the-art theoretical results presented in [13,22]. A tandem queuing system is simulated where the hop counts is *L*. The arrival process follows the Poisson with average rate λ. The analysis method proposed in [22] can only analyze the E2E delay performance for the tandem queuing system with nodes supplying homogeneous service processes. Therefore, it is set that the service processes of nodes obey Bernoulli distributions with successful probability ps and service rate *C*. In each simulation, the E2E delay of $10^{6}$ slots is recorded and the E2E delay violation probability is calculated. The simulated E2E delay violation probabilities are shown in the box plot. The differences between theoretical values of upper bounds and average simulated values are summarized in Table 4 and Table 5, respectively.

With different hop counts, the martingale-based upper bounds of E2E delay violation probability are closer to the average simulated results than those obtained by the other methods, indicating tighter bounds. The method in [13] based on the SNC theory is used to analyze the E2E delay of the tandem queuing system, where the arrival process follows a Gaussian distribution and the service process of each node is a constant. This result is relatively conservative for estimating the delay violation behavior, which could trigger the resource waste when the SNC-based upper bound guides bandwidth demands capture. The martingale-based method proposed in [22] focuses on the Markov-modulated arrival and service processes, and the service processes should be homogeneous, which are unable to analyze the E2E delay performance for the tandem queuing system with the i.i.d. arrival and service processes.

**Figure 4:**
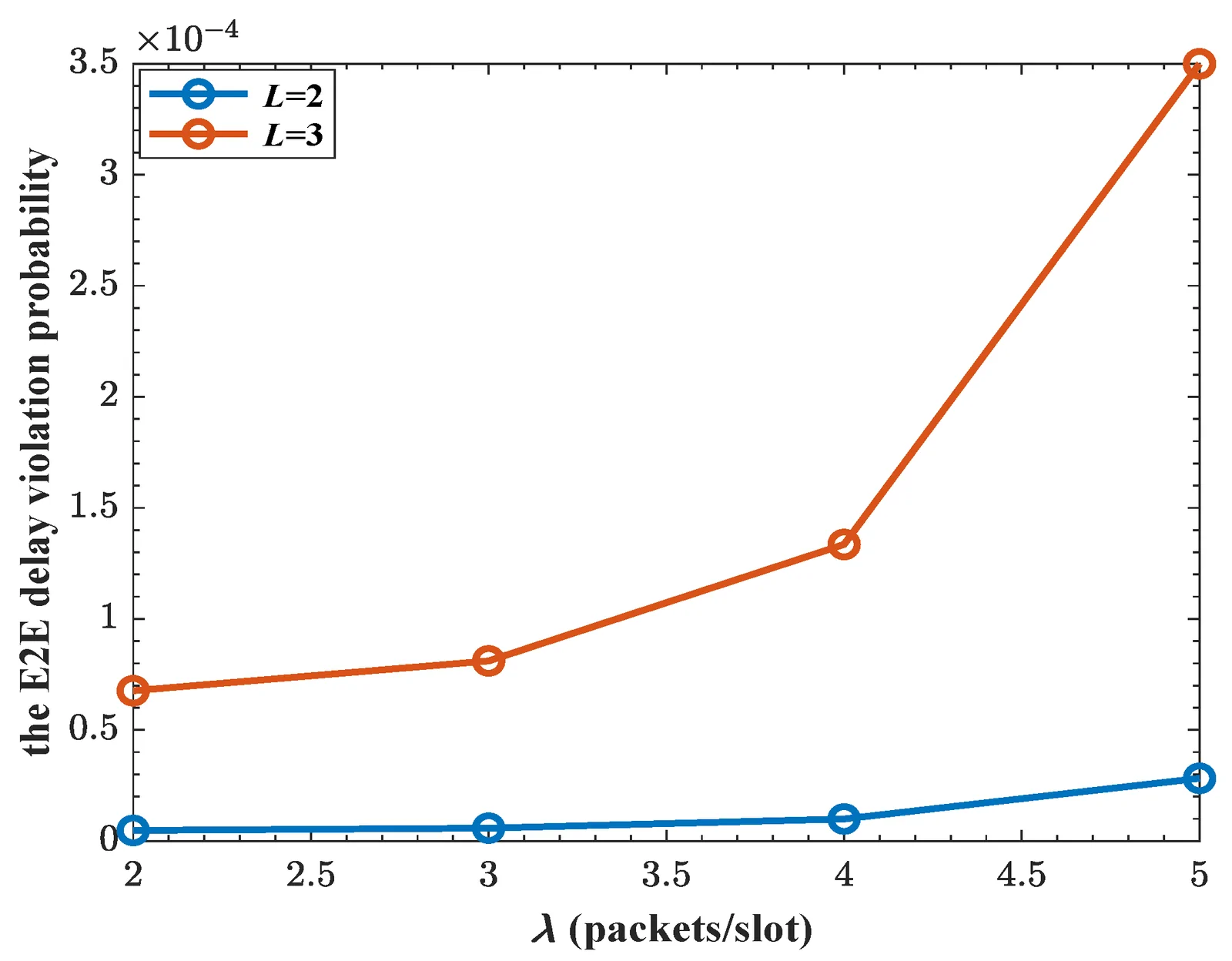
The E2E delay violation probability vs. arrival load, where ps=0.9, $C^{1}=10$ packets/slot, $C^{2}=10$ packets/slot, $C^{3}=10$ packets/slot and $W_{\max}=8$ slots.

**Figure 5:**
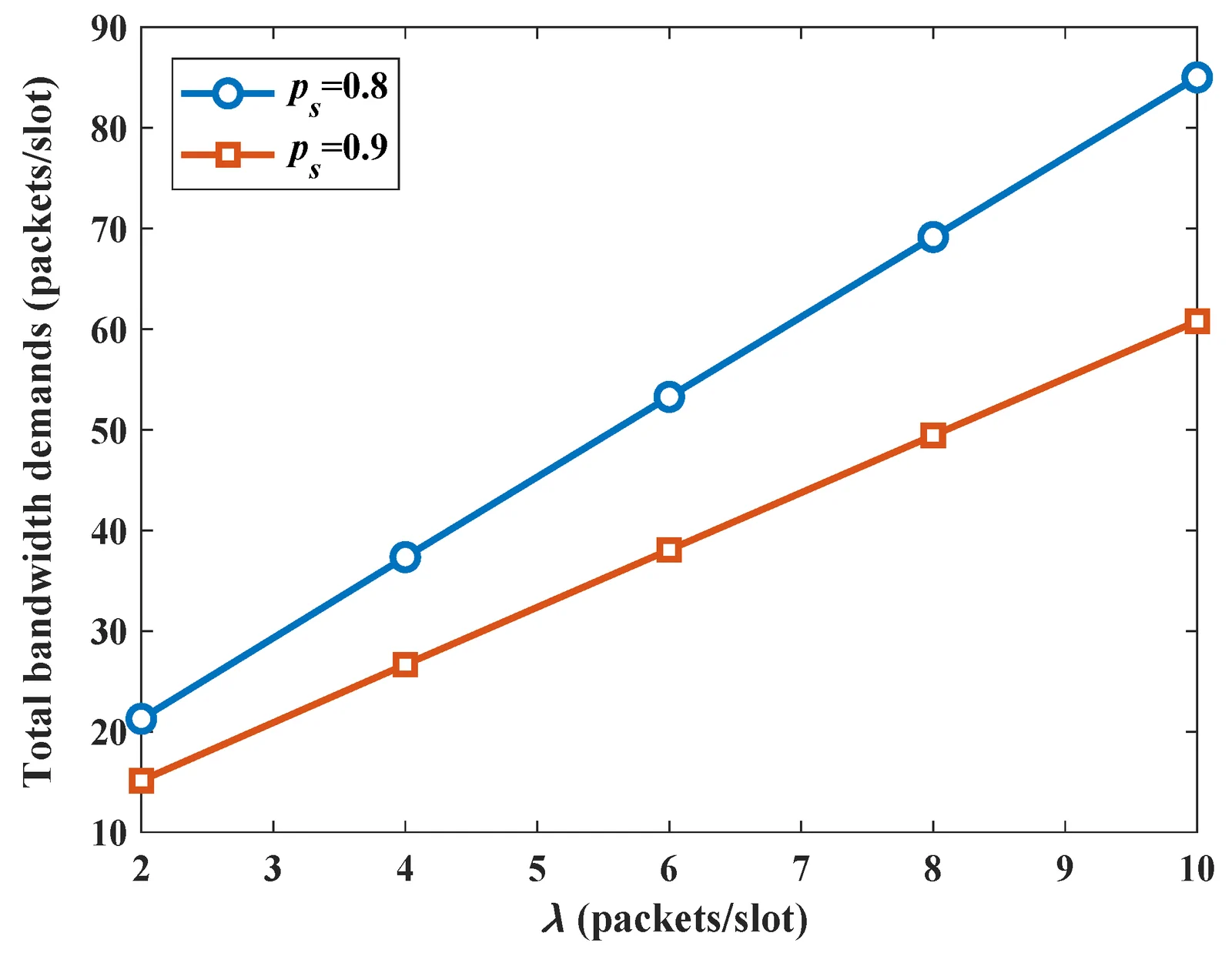
The total bandwidth demands vs. arrival load, where $W_{\max}=8$ slots.

**Figure 6:**
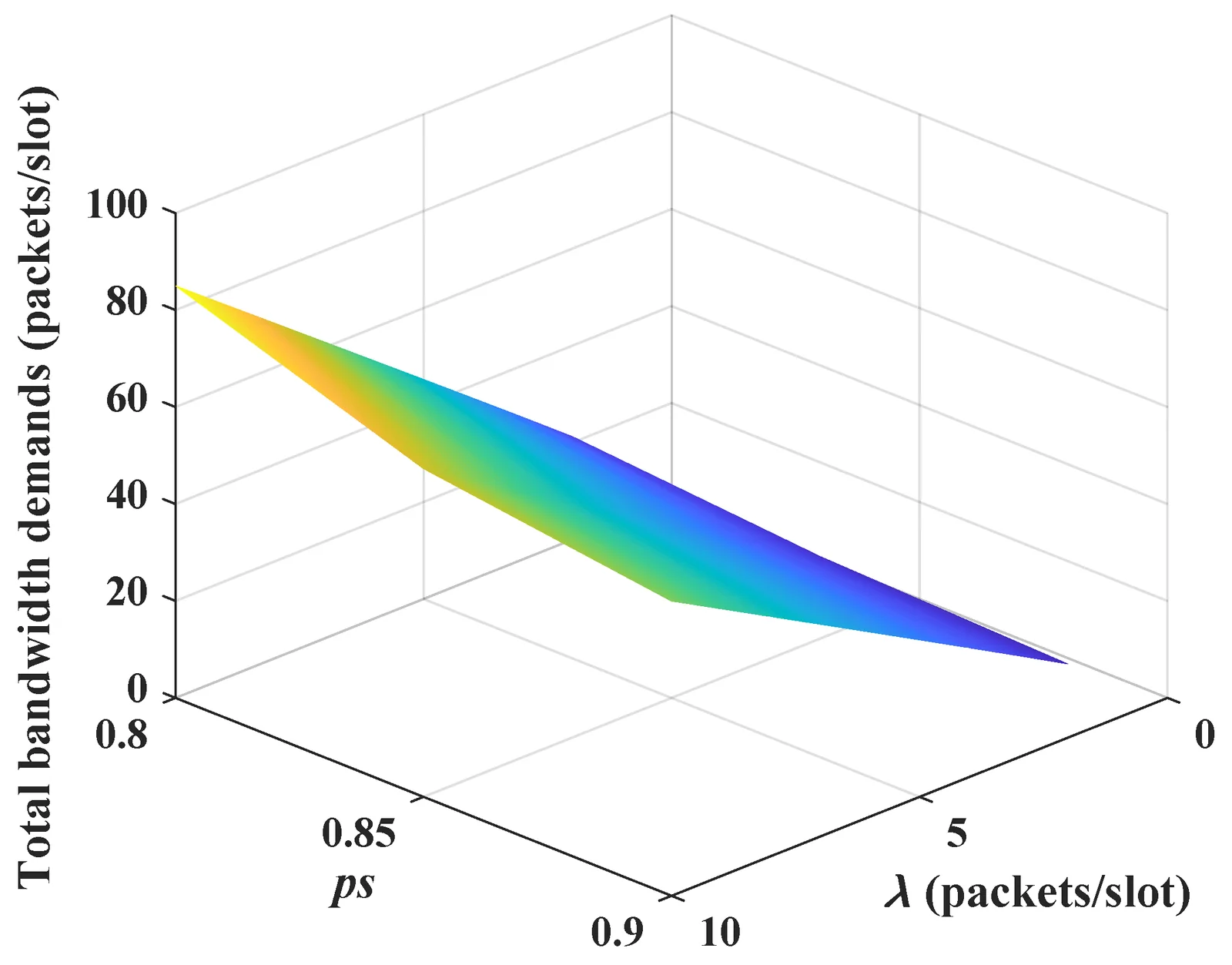
The total bandwidth demands vs. arrival load and the successful access probability ps, where $W_{\max}=8$ slots.

The impact factors on the E2E delay performance and the bandwidth demands are explored theoretically. The E2E delay violation probability versus the arrival load and the hop counts are shown in Figure 4. The number of hops is L=2 or L=3. The service model of the first node follows the Bernoulli distribution with the successful service probability ps and service rate $C^{1}$, while the one of the second node is a constant $C^{2}$. If L=3, the service process of the last node is also a constant $C^{3}$. As observed, a heavier system load and a higher hop count both damage the delay performance. With the arrival load increasing, the delay violation probability rises sharply when the E2E traffic travels more nodes. The E2E delay violation probability increases exponentially, not linearly. For the E2E traffic with strict E2E delay QoS requirements, the length of the E2E path should be controlled. Otherwise, more bandwidth resources should be consumed to guarantee the QoS requirements. In Figure 5 and Figure 6, the total bandwidth demands of the parallel system are obtained by performing the BDC-RD scheme. A parallel system consists of two E2E paths with different hop counts (L=2, L=3) is considered. It can be seen that the demanded service rate must surge to counteract the impact of the decline in success access probability and the increasing arrival load on delay performance, so that the uniform E2E delay QoS requirements can be provisioned.

**Figure 7:**
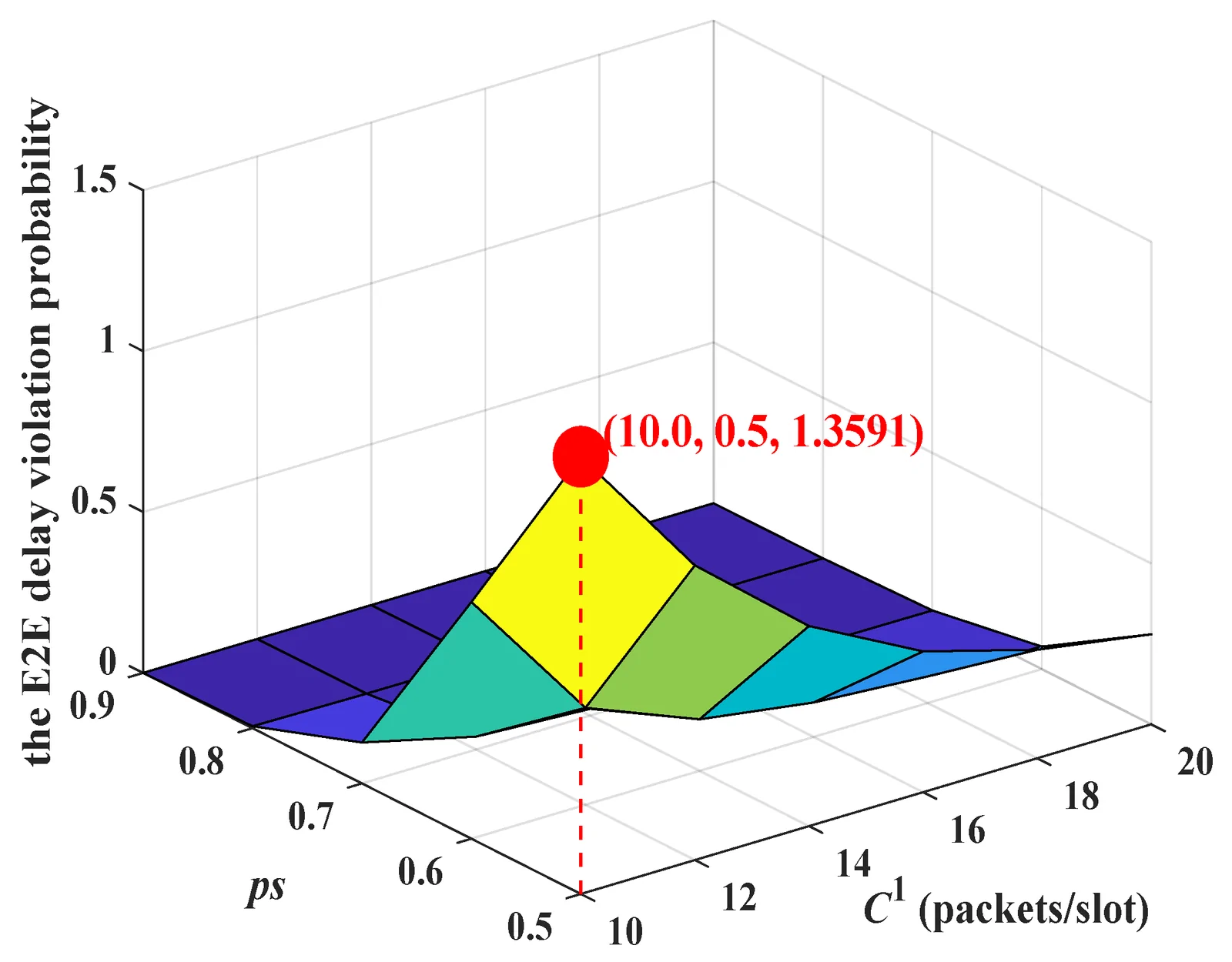
The E2E delay violation probability vs. ps and $C^{1}$, where λ=5 packets/slot, $C^{2}=10$ packets/slot and $W_{\max}=8$ slots.

**Figure 8:**
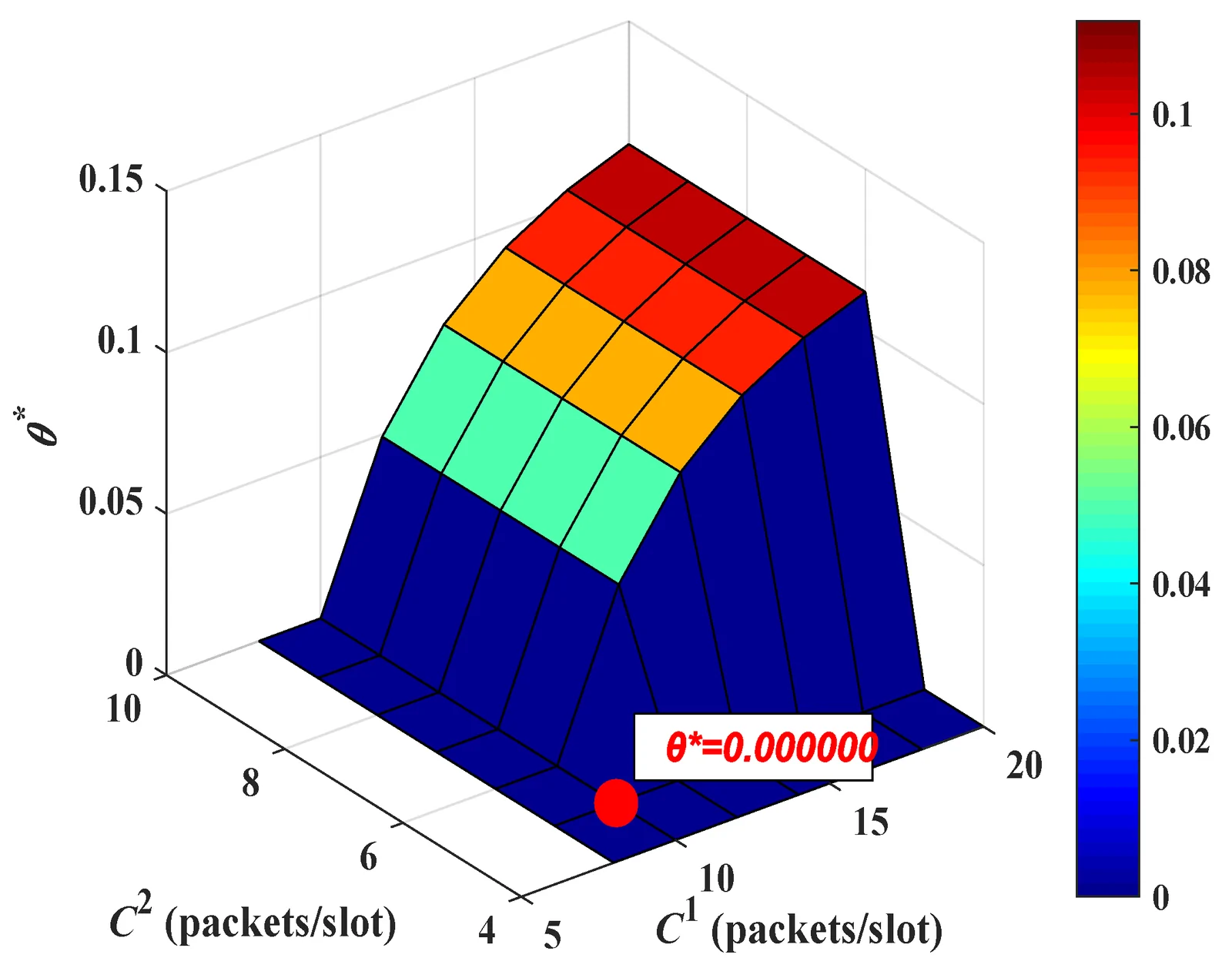
The service index $\theta^{*}$ vs. $C^{1}$ and $C^{2}$, where λ=5 packets/slot, ps=0.9 and $W_{\max}=8$ slots.

**Figure 9:**
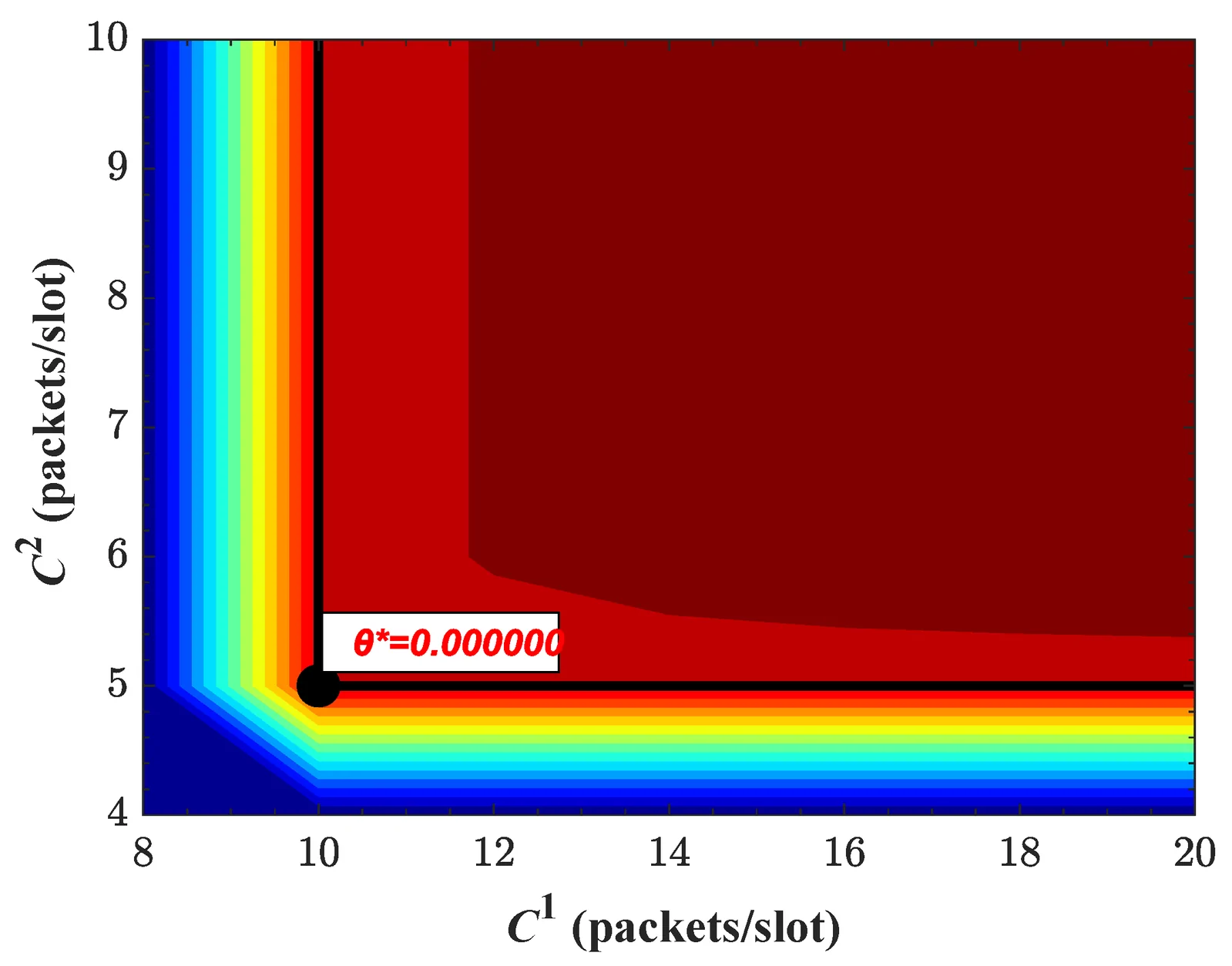
The limitation of service index $\theta^{*}$ vs. $C^{1}$ and $C^{2}$, where λ=5 packets/slot, ps=0.9 and $W_{\max}=8$ slots.

The influence of the service configuration is analyzed, and the results are shown in Figure 7, Figure 8 and Figure 9. The tandem queuing system possesses two hops. The service process of the first node is modeled as the Bernoulli distribution with the successful service probability ps and service rate $C^{1}$. The second node provides a constant service rate $C^{2}$. In Figure 7, the constant service rate of node 2 is fixed. The enhancement of ps or $C^{1}$ both improve the E2E delay performance. By contrast, the decline of the successful service probability is more fatal for QoS guarantee than the reduction in the service rate. The limitation of the service configuration in each node should be focused on. As shown in Figure 7, the average arrival rate of the E2E traffic is λ=5 packets/slot. The average service rate of the first node is $C^{av1}=ps\times C^{1}$. When the average service rate $C^{av1}$ is lower than the average arrival rate, the theoretical result of E2E delay violation probability $P\{W\left(t\right)\geq W_{\max}\}\geq 1$, which implies that the buffer of this node could overflow from a long time scale and this E2E path will break. Further, the proposed martingale-based analysis framework is invalid.

The defined service index can reveal the impacts of service on system performance more clearly. In Definition 4, the service index of the E2E path *l* is $\theta_{l}^{*}$, which is limited to $\theta_{l}^{*}\geq 0$ so that the theoretical upper bound shown in Theorem 1 holds. The service index (abbreviated to $\theta^{*}$) versus the service configuration in each node is presented in Figure 8 and Figure 9. The E2E delay violation probability is negatively correlated with $\theta^{*}$. $\theta^{*}$ measures the service ability of the tandem queuing system. A larger service index implies the E2E path could support higher reliability requirements. In a tandem queuing system, the lower limit of service rates is that the average service rate is larger than the average arrival rate of each node. As shown in Figure 9, the upper right corner marked by the black line is the feasible range of the current tandem system. As long as one node does not meet this constraint, the entire system will be unable to operate normally.

**Figure 10:**
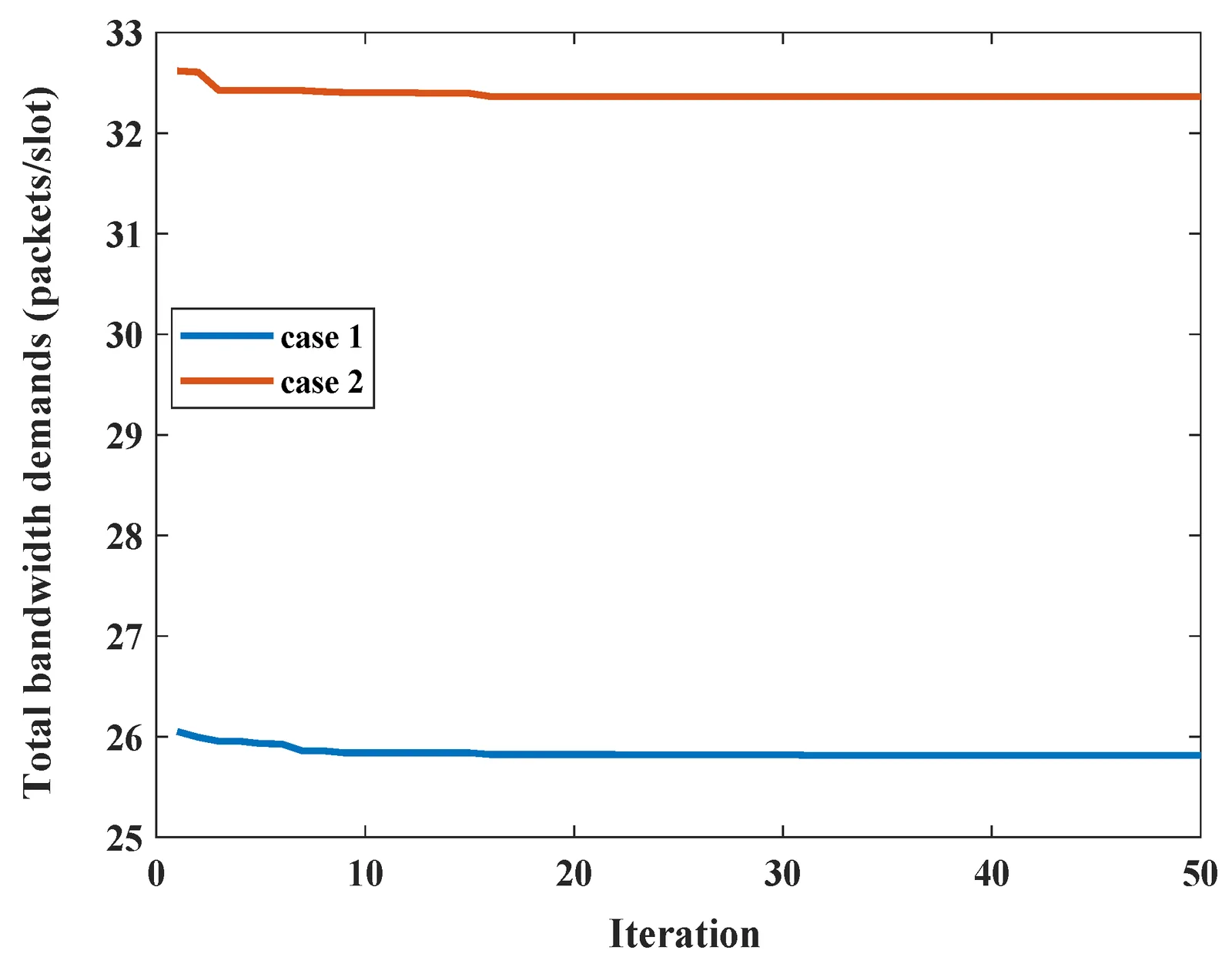
The evolution of total bandwidth demands in the BDC-RD scheme, where λ=5 packets/slot, ps=0.9 and $W_{\max}=8$ slots.

**Figure 11:**
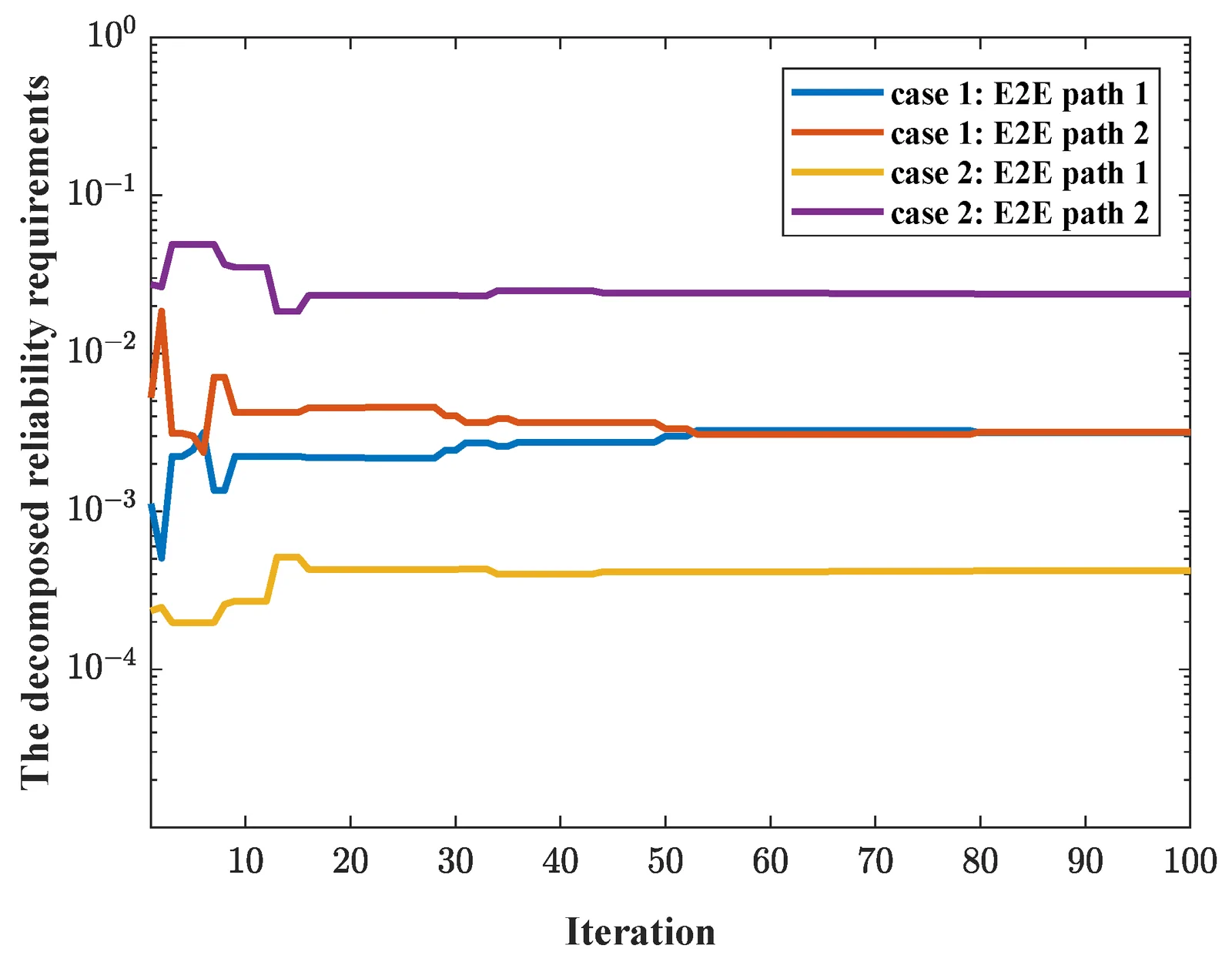
The evolution of the decomposed E2E delay reliability requirement of each E2E path in the BDC-RD scheme, where λ=5 packets/slot, ps=0.9 and $W_{\max}=8$ slots.

The stability and convergence of the designed BDC-RD scheme are verified, and the results are presented in Figure 10 and Figure 11. We consider two parallel transmission patterns. In case 1, the parallel system consists of two E2E paths with the same hop counts (L=2, L=2), and case 2 is that the system consists of two E2E paths with different hop counts (L=2, L=3). Figure 10 shows the total bandwidth demands of these two systems with the evolution of the number of iterations. The objective value of the BDC-RD scheme becomes stable within approximately 50 iterations. The parallel system possessing more hop counts must consume more bandwidth resources to achieve the QoS provisioning.

To further assess the quality of the PSO feasible solution, we additionally performed a branch-and-bound search for Problem (26). A deterministic spatial branch-and-bound method can provide lower and upper bounds on the globally optimal objective value for nonconvex problems [35]. The branch-and-bound search is used only for numerical validation and is not part of the proposed BDC-RD scheme. By exploiting the monotonic relationships between the service rate, total bandwidth demand, and delay violation probability bound, lower bounds on the objective value can be constructed for different search regions. The branch-and-bound method divides the search space into smaller regions and evaluates their lower bounds, while the best feasible solution found so far provides an upper bound. If the lower bound of a region is no smaller than the current upper bound, that region can be excluded because it cannot yield a better solution. The remaining regions are further divided to progressively reduce the optimality gap.

For this comparison, 50 independent PSO runs are performed with different random initializations. Let $B_{\mathrm{LB}}$ and $B_{\mathrm{UB}}$ denote the lower and upper bounds on the globally optimal objective value obtained by the branch-and-bound search, respectively. $B_{\mathrm{PSO}}$ denotes the minimum objective value among the feasible solutions obtained from these 50 independent PSO runs. The relative optimality-gap upper bound $\Delta_{\mathrm{PSO}}^{\mathrm{UB}}$ is calculated as

(42) $$\Delta_{\mathrm{PSO}}^{\mathrm{UB}}=\frac{B_{\mathrm{PSO}}-B_{\mathrm{LB}}}{B_{\mathrm{LB}}}\times 100\%.$$

$\Delta_{\mathrm{PSO}}^{\mathrm{UB}}$ represents an upper bound on the relative optimality gap between the PSO solution and the global optimum. A smaller value indicates a tighter bound on the possible deviation of the PSO solution from the global optimum.

As shown in Table 6, the small difference between $B_{\mathrm{LB}}$ and $B_{\mathrm{UB}}$ indicates that the global optimum is tightly bounded. The corresponding $\Delta_{\mathrm{PSO}}^{\mathrm{UB}}$ values are only 0.000230% and 0.000116% for case 1 and case 2, respectively, indicating that the PSO solutions are very close to the global optimum under the considered settings.

The process of the reliability requirement decomposition is presented in Figure 11. For each case mentioned above, every E2E path is allocated reliability requirement to carry so that the reliability of the parallel system can meet $P\{W\left(t\right)\geq W_{\max}\}\leq \zeta$. The BDC-RD scheme can complete the reliability decomposition within 100 iterations, which proves its stability and convergence. As observed, for case 1, the E2E paths with the same hop counts need to guarantee the uniform reliability requirements, which are both $\zeta^{\frac{1}{2}}$. For case 2, due to the different hop counts, the shorter E2E path should carry more strict reliability requirement than the other, which facilitates a reduction in total bandwidth demands. In the martingale-based E2E delay performance analysis framework, the impacts of hop counts have been considered in the theoretical upper bound, which contributes to decompose the reliability requirements reasonably and minimize the total bandwidth demands.

**Figure 12:**
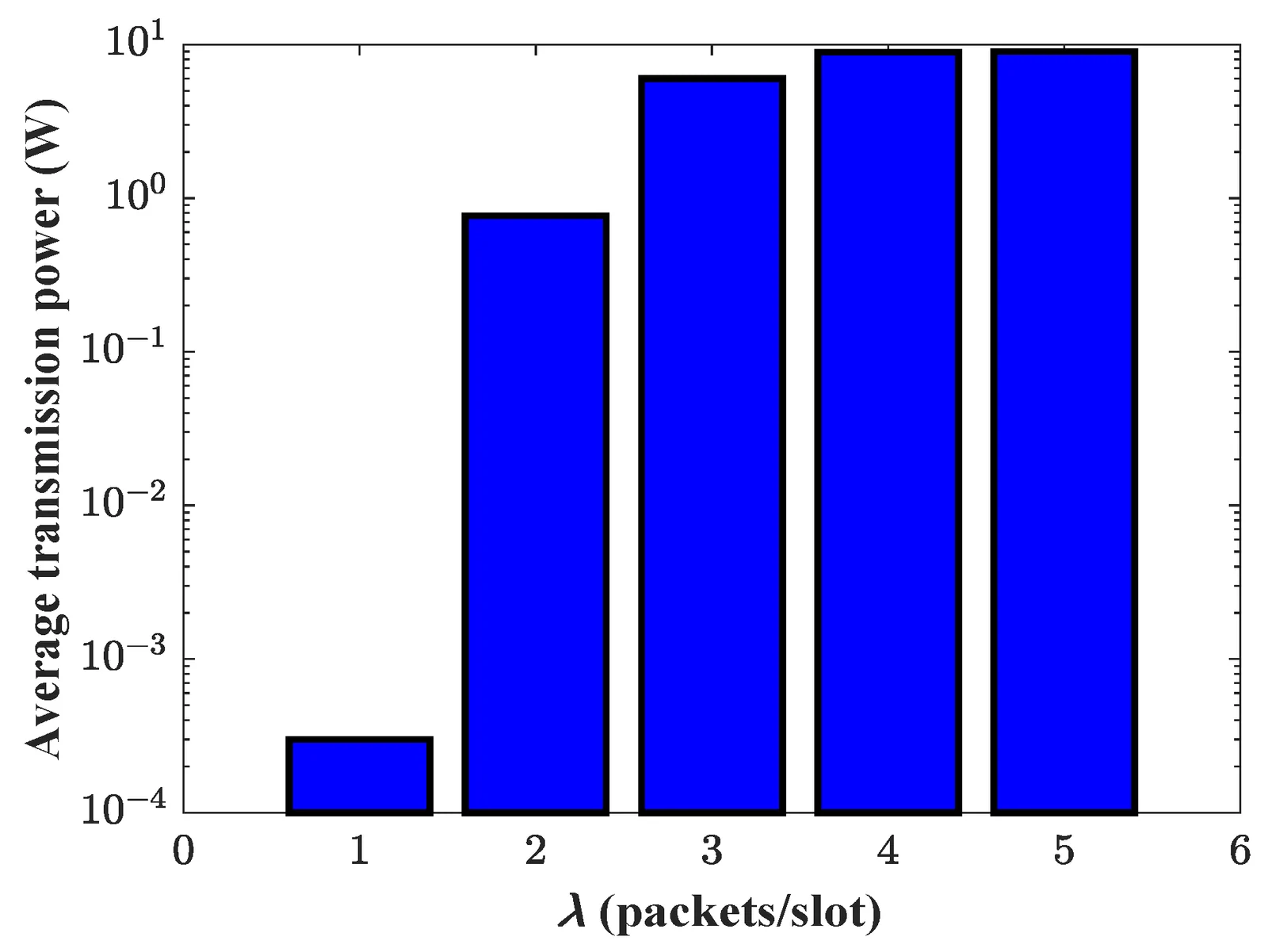
The average transmission power vs. arrival load, where ps=0.9 and $W_{\max}=8$ slots.

The influence factors of the transmission power consumption are analyzed. In Figure 12, the parallel system of case 1 is considered. By the BDC-RD scheme, the demanded service rates of these two E2E paths in the wireless access network are determined, which guide the physical layer resource allocation algorithm. The observation time window is $10^{5}$ time slots. The average transmission power of the parallel system is simulated with the arrival load. Following the same line as the bandwidth demands, the power consumption also surges with the heavier arrival load. It is worth mentioning that the E2E delay QoS requirement is a statistical probability. Leveraging the BDC-RD scheme, it is mapped as confirmable bandwidth demands, which could guide the physical layer resource allocation naturally. Thus, the transmission power allocation algorithm is designed under the statistical E2E delay QoS constraints.

**Figure 13:**
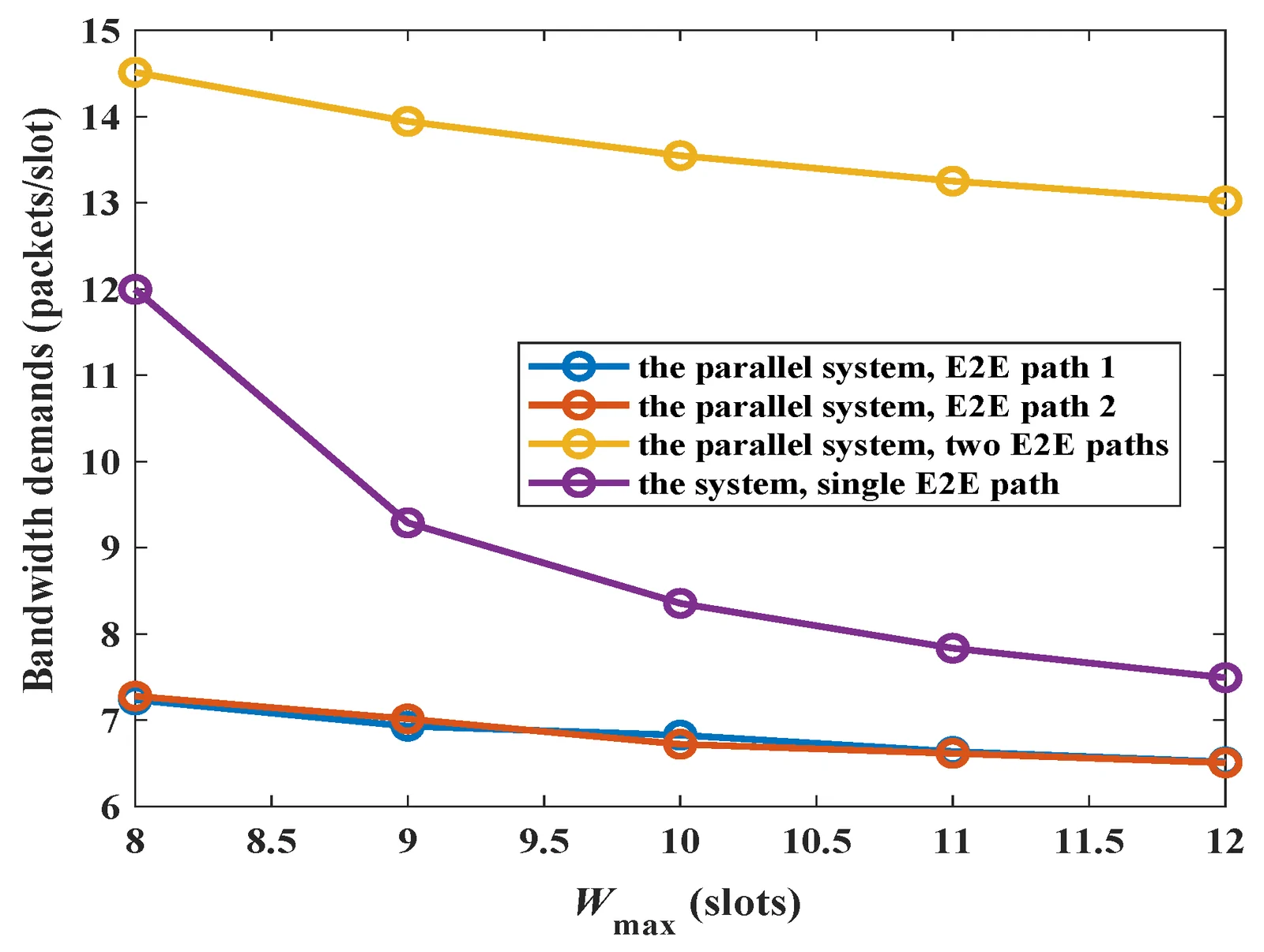
$W_{\max}$ vs. the bandwidth demands, where λ=5 packets/slot, ps=0.9 and L=2. The parallel system consists of two E2E paths. The hop counts are both 2.

**Figure 14:**
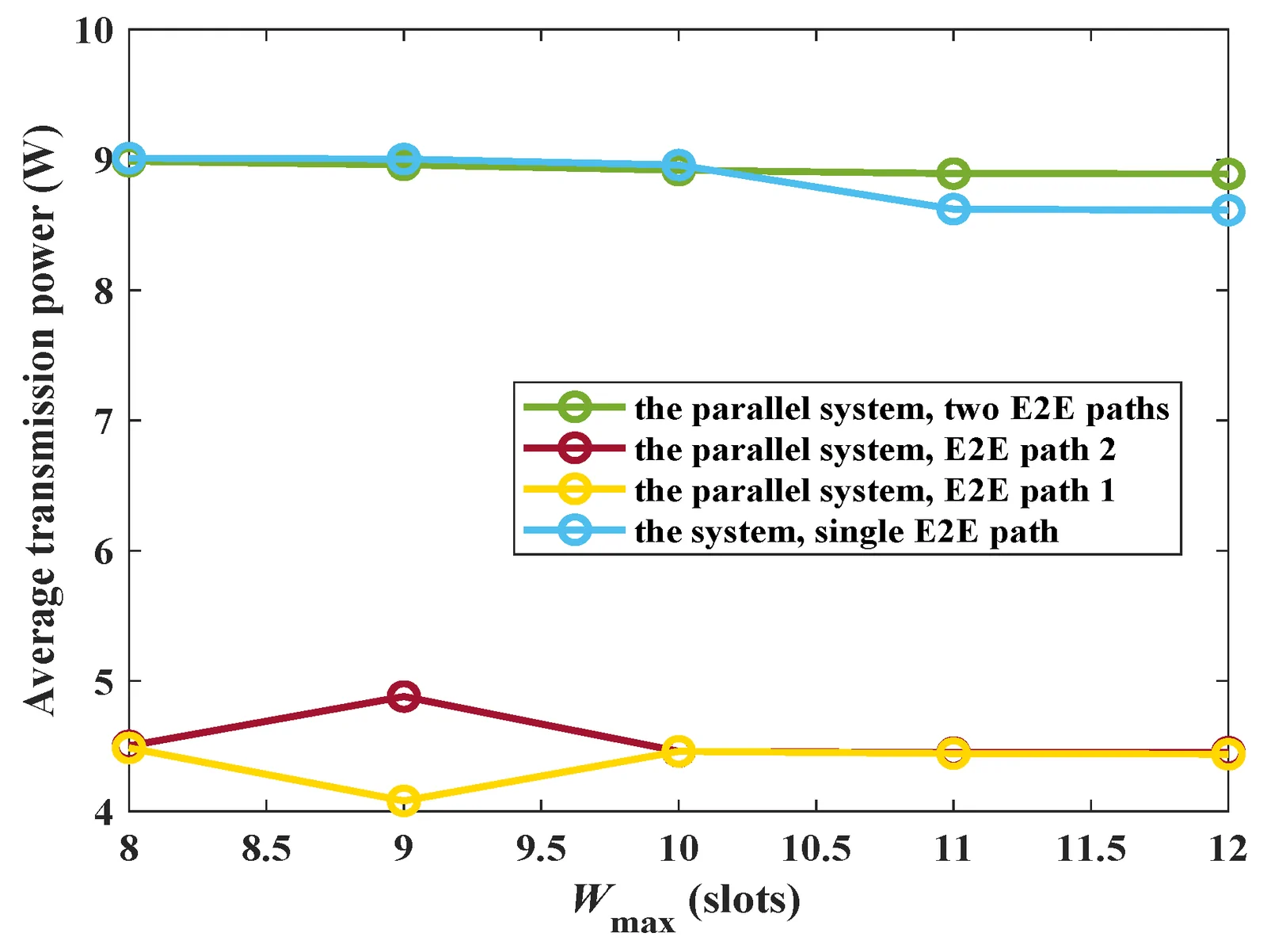
$W_{\max}$ vs. the transmission power, where λ=5 packets/slot, ps=0.9 and L=2. The parallel system consists of two E2E paths. The hop counts are both 2.

In Figure 13 and Figure 14, the comparison between the hot-backup transmission pattern and the single path transmission pattern is presented. For the parallel system, the total bandwidth demands are captured by the BDC-RD scheme, and the required bandwidth of each E2E path is also recorded.

**Figure 15:**
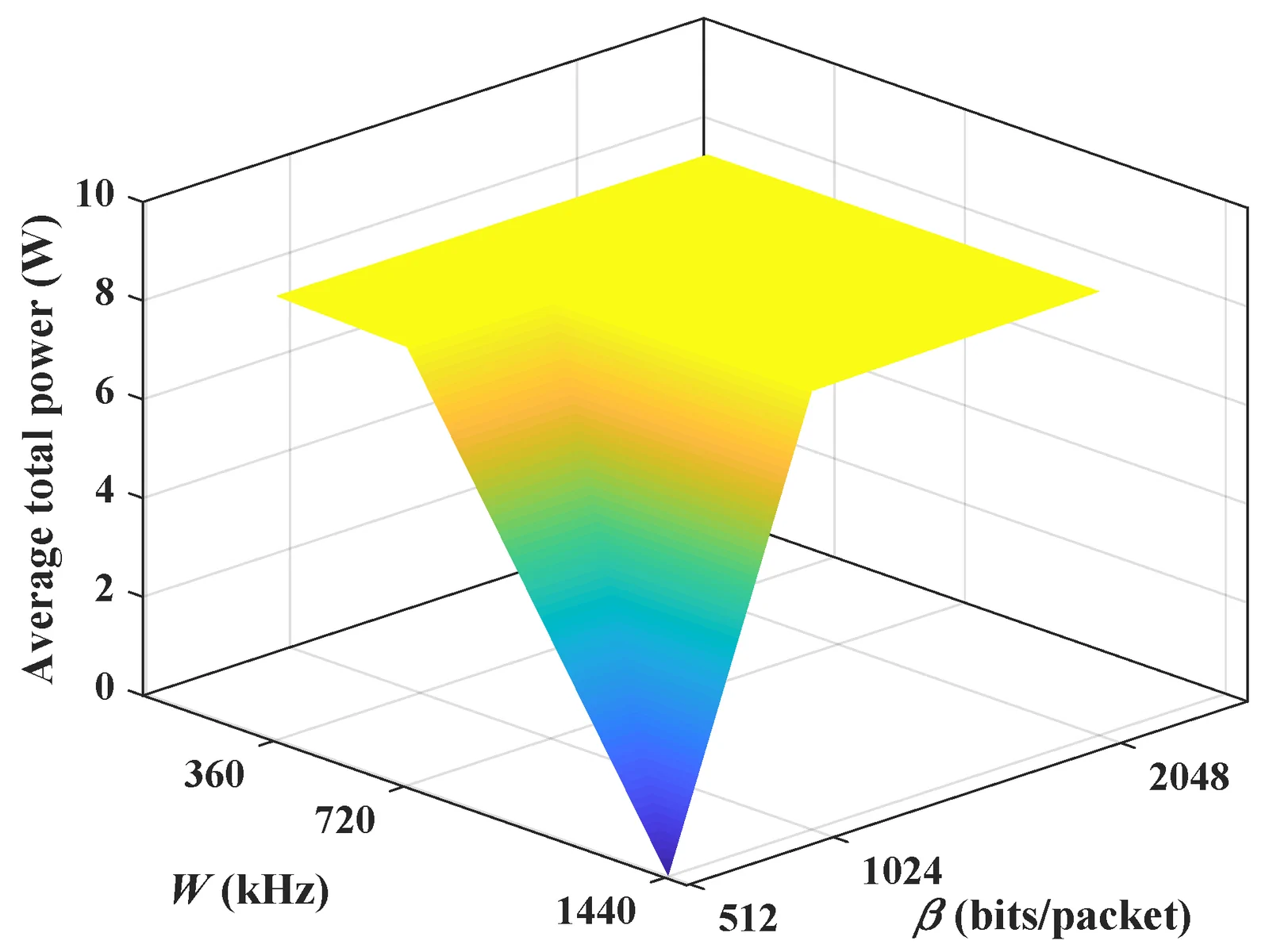
The impacts of the physical layer parameters configuration, where λ=5 packets/slot, ps=0.9. The parallel system consists of two E2E paths. The hop counts are both 2.

As observed, the parallel system indeed incurs higher resource overhead than the single path transmission scheme to guarantee the same QoS requirement. However, the service rate required on each path is substantially lower than that in the single path case, which is beneficial for load balancing across the entire network, particularly when more URLLC terminals need to be carried. For both the DUs in wireless access networks and the forwarding nodes, the substantially reduced bandwidth demand on each individual path enables the system to better utilize scattered idle resources across the network, thereby mitigating resource fragmentation and enhancing overall scheduling flexibility. Figure 14 illustrates the transmission power consumption by applying the proposed physical layer resource allocation algorithm to the setup of Figure 13. It can be seen that, although the bandwidth requirements vary considerably across different cases, the corresponding impact on the physical layer transmission power remains relatively limited. This is mainly attributed to the fact that other physical layer parameter configurations can effectively maintain the stability of power allocation. To further investigate this issue, Figure 15 presents the influence of physical-layer parameter settings on the power allocation performance. According to the numerology design of 5G-New Radio, a resource block (RB) has the bandwidth equal or 2, 4, 8, or 16 times of 180 kHz and the time duration equal or 2, 4, 8, or 16 times smaller than 0.5 ms [36]. The influence of system bandwidth and the length of a packet on the required power is shown. The results indicate that a wider bandwidth of a RB and a shorter packet length lead to lower power consumption. Since the bandwidth of a RB is closely related to the duration of a time slot, a wider bandwidth of a RB facilitates QoS provisioning. However, the extremely short time slot may introduce considerable challenges in terms of system control and management.

## 8. Conclusions

In this paper, a novel E2E delay QoS provisioning strategy is proposed based on martingale theory. The martingales related to arrival and service processes are defined, and the martingale parameters are determined by considering the heterogeneity of the stochastic characteristics. The bias scale functions are introduced to model the statistical features of accumulated arrival and service processes. A service index is defined in the martingale domain to characterize the service capability of a tandem queuing system for a given arrival process. Based on the above, a tight upper bound of the E2E delay violation probability is derived. Guided by the martingale-based theoretical result, the BDC-RD scheme decouples the statistical E2E delay QoS requirement into the demanded service rates of the involved nodes and decomposes the reliability requirement across multiple E2E paths. Under the bandwidth demands constraints, a physical layer resource allocation algorithm is designed. The closed form of the transmission power is obtained by the Lagrange gradient algorithm, which could guarantee the E2E delay QoS requirements. The simulations verify the effectiveness and superiority of the proposed reliability provisioning scheme. The tightness of the martingale-based upper bound is demonstrated by comparison with the state-of-the-art results. The relative optimality-gap upper bounds of 0.000230% and 0.000116% further indicate that the PSO solutions are very close to the global optimum under the considered settings.

Future work will further extend the proposed framework to more general network conditions. First, the i.i.d. and independence assumptions of the arrival and service processes will be relaxed to consider temporally correlated traffic and service processes. Second, more dynamic network conditions, such as time-varying service capabilities and network states, will be considered to further evaluate the applicability of the proposed E2E delay analysis framework. The possible correlation among parallel E2E paths due to shared physical nodes or imperfect logical isolation will also be considered in future work. In such cases, the reliability assessment will be further extended from the current product-form model to a joint-distribution-based framework. In addition, the proposed framework will be extended to multi-user scenarios, where joint reliability decomposition and resource allocation will be further studied.

## Figures and Tables

**Table 1:** The main symbols and notations.

Notations	Symbols
Time slot	*t*
The set of E2E paths	L
The set of nodes in the E2E path *l*	$H_{l}$
The transmission power of path *l* at time slot *t*	$p_{l}^{1}\left(t\right)$
The forwarding rate of router node *h*	$C_{h}$
The successful access probability	$p_{s}$
The arrival process in E2E path *l*	$\left\{a_{l}\left(t\right),t\geq 0\right\}$
The accumulated arrival process in E2E path *l*	$\left\{A_{l}\left(m,n\right),n\geq m\geq 0\right\}$
The service process of node *h* in E2E path *l*	$\left\{s_{l}^{h}\left(t\right),t\geq 0\right\}$
The accumulated service process of node *h* in E2E path *l*	$\left\{S_{l}^{h}\left(m,n\right),n\geq m\geq 0\right\}$
The accumulated departure process in E2E path *l*	$\left\{D_{l}\left(m,n\right),n\geq m\geq 0\right\}$
The E2E delay in E2E path *l* at slot *t*	$W_{l}\left(t\right)$
The E2E delay threshold	$W_{max}$
The EB of arrival process $\left\{a_{l}\left(t\right),t\geq 0\right\}$	$EB_{l}\left(\theta \right)$
The martingale related to arrival	$M_{a}^{l}\left(m,t\right)$
The EC of service process $\left\{s_{l}^{h}\left(t\right),t\geq 0\right\}$	$EC^{l,h}\left(\theta \right)$
The martingale related to service	$M_{s}^{l,h}\left(m,t\right)$
The service index	$\theta_{l}^{*}$
The bias scale function of arrival	$\Psi_{a}^{l}\left(m,t\right)$
The bias scale function of service	$\Psi_{s}^{l,h}\left(m,t\right)$
The bandwidth demands of node *h* in E2E path *l*	$C_{l}^{h}$

**Table 2:** The main abbreviations.

Full Names	Abbreviations	Full Names	Abbreviations
end-to-end	E2E	sparse code multiple access	SCMA
Ultra-Reliable Low Latency Communications	URLLCs	user equipment	UE
bandwidth demands capture and reliability decomposition	BDC-RD	next-generation NodeB	gNB
particle swarm optimization	PSO	centralized unit	CU
quality of service	QoS	distributed unit	DU
radio access network	RAN	active antenna unit	AAU
independent identical distributed	i.i.d.	Common Public Radio Interface	CPRI
stochastic network calculus	SNC	medium access control	MAC
effective bandwidth	EB	radio resource control	RRC
effective capacity	EC	packet data convergence protocol	PDCP
Non-Orthogonal Multiple Access	NOMA	virtualized baseband unit	vBBU
Machine-Type Communication	MTC	user plane function	UPF

**Table 3:** Commonly used simulation parameters.

Parameters	Values
The average load of arrival traffic, λ	5 packets/slot
The length of a packet, β	512 bits
The latency threshold, $W_{\max}$	1 ms
The reliability requirement of E2E latency within $W_{\max}$, 1−ζ	99.999%
The bandwidth allocated to a DU, *W*	2.16 MHz
The duration of a time slot, α	0.125 ms
The noise power spectral density, $\sigma^{2}$	−174 dBm/Hz
The maximum transmission power of the UE, $P^{\max}$	5 W
The successful transmission probability, $p_{s}$	0.9

**Table 4:** The gaps between theoretical upper bounds and average simulated delay violation probability with L=2.

	$W_{\max}$ (Slots)	5	6	7	8	9	10
Method							
Average simulated results		$9.82\times 10^{-3}$	$1.52\times 10^{-3}$	$1.88\times 10^{-4}$	$3.10\times 10^{-5}$	$5.33\times 10^{-6}$	0
Martingale-based		$1.36\times 10^{-2}$	$2.95\times 10^{-3}$	$6.12\times 10^{-4}$	$1.23\times 10^{-4}$	$2.39\times 10^{-5}$	$4.56\times 10^{-6}$
in [13]		$8.50\times 10^{-1}$	$2.90\times 10^{-1}$	$5.60\times 10^{-2}$	$1.08\times 10^{-2}$	$2.09\times 10^{-3}$	$4.03\times 10^{-4}$
in [22]		$2.41\times 10^{-4}$	$4.56\times 10^{-5}$	$8.61\times 10^{-6}$	$1.63\times 10^{-6}$	$3.08\times 10^{-7}$	$5.81\times 10^{-8}$

**Table 5:** The gaps between theoretical upper bounds and average simulated delay violation probability with L=3.

	$W_{\max}$ (Slots)	5	6	7	8	9	10
Method							
Average simulated results		$8.93\times 10^{-2}$	$1.90\times 10^{-2}$	$3.97\times 10^{-3}$	$3.98\times 10^{-4}$	$1.66\times 10^{-4}$	$5.00\times 10^{-6}$
Martingale-based		$1.29\times 10^{-1}$	$3.29\times 10^{-2}$	$7.84\times 10^{-3}$	$1.78\times 10^{-3}$	$1.86\times 10^{-4}$	$8.13\times 10^{-5}$
in [13]		>1	>1	>1	$8.11\times 10^{-1}$	$1.57\times 10^{-1}$	$3.02\times 10^{-2}$
in [22]		$2.41\times 10^{-4}$	$4.56\times 10^{-5}$	$8.61\times 10^{-6}$	$1.63\times 10^{-6}$	$3.08\times 10^{-7}$	$5.81\times 10^{-8}$

**Table 6:** Numerical validation of the PSO solution quality.

Scenario	[$B_{\mathrm{LB}}$, $B_{\mathrm{UB}}$] (Packets/Slot)	$B_{\mathrm{PSO}}$ (Packets/Slot)	$\Delta_{\mathrm{PSO}}^{\mathrm{UB}}$ (%)
Case 1 (2,2)	[25.812510,25.812536]	25.812569	0.000230
Case 2 (2,3)	[32.362618,32.362651]	32.362656	0.000116

## Data Availability

The data presented in this study are available upon request from the corresponding authors.
